# Single-cell transcriptomic analysis of decidual immune cell landscape in the occurrence of adverse pregnancy outcomes induced by *Toxoplasma gondii* infection

**DOI:** 10.1186/s13071-024-06266-w

**Published:** 2024-05-10

**Authors:** Tianyi Fu, Xiaohui Wang, Xiaoyue Zhao, Yuzhu Jiang, Xianbing Liu, Haixia Zhang, Yushan Ren, Zhidan Li, Xuemei Hu

**Affiliations:** 1 Department of Immunology, Binzhou Medical University, Yantai, 264003 Shandong People’s Republic of China; 2 Department of Clinical Psychology, Yantai Affiliated Hospital of Binzhou Medial University, Yantai, 264100 Shandong People’s Republic of China

**Keywords:** Single-cell transcriptomic, Maternal–fetal tolerance, Abnormal pregnancy, *Toxoplasma gondii*, Decidual immune cell

## Abstract

**Background:**

*Toxoplasma gondii* is an obligate intracellular parasite that can lead to adverse pregnancy outcomes, particularly in early pregnancy. Previous studies have illustrated the landscape of decidual immune cells. However, the landscape of decidual immune cells in the maternal–fetal microenvironment during *T. gondii* infection remains unknown.

**Methods:**

In this study, we employed single-cell RNA sequencing to analyze the changes in human decidual immune cells following *T. gondii* infection. The results of scRNA-seq were further validated with flow cytometry, reverse transcription-polymerase chain reaction, western blot, and immunofluorescence staining.

**Results:**

Our results showed that the proportion of 17 decidual immune cell clusters and the expression levels of 21 genes were changed after *T. gondii* infection. Differential gene analysis demonstrated that *T. gondii* infection induced the differential expression of 279, 312, and 380 genes in decidual NK cells (dNK), decidual macrophages (dMφ), and decidual T cells (dT), respectively. Our results revealed for the first time that several previously unknown molecules in decidual immune cells changed following infection. This result revealed that the function of maternal–fetal immune tolerance declined, whereas the killing ability of decidual immune cells enhanced, eventually contributing to the occurrence of adverse pregnancy outcomes.

**Conclusions:**

This study provides valuable resource for uncovering several novel molecules that play an important role in the occurrence of abnormal pregnancy outcomes induced by *T. gondii* infection.

**Graphical Abstract:**

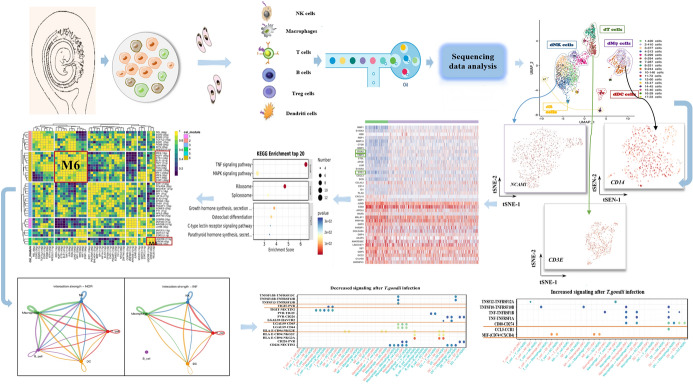

**Supplementary Information:**

The online version contains supplementary material available at 10.1186/s13071-024-06266-w.

## Background

*Toxoplasma gondii* is an obligate intracellular protozoan parasite with an extensive distribution [1]. Pregnant women infected with *T. gondii* can experience miscarriages, premature deliveries, stillbirths, and other adverse pregnancy outcomes during first trimester [2]. The microenvironment at the maternal–fetal interface plays an important role in maintaining normal pregnancy [3]. It is composed of decidual immune cells, cytokines, and enzymatic factors [4]. The function of decidual immune cells in maternal–fetal tolerance is mainly dependent on their expression of several types of inhibitory molecules [5]. Our previous studies have demonstrated that *T. gondii* infection results in the dysfunction of several immune cells, such as dNK, dMφ, and dTreg, thereby contributing to abnormal pregnancy outcomes [6–8]. Further studies have reported that *T. gondii* infection can affect the expression levels of some inhibitory molecules (leukocyte immunoglobulin-like receptor B4, inhibitory receptor T-cell immunoglobulin and mucin domain 3, and B7 homolog 4, etc.) in these immune cells and then regulate the expression of functional molecules (IL-10, TGF-β, and TNF-α etc.) [9–11]. These effects eventually lead to the dysfunction of immune cells. However, the numerous unknown immune molecules in decidual immune cells that may participate in the above process and contribute to adverse pregnancy outcomes during *T. gondii* infection need to be evaluated.

As a revolutionary technology, single-cell RNA sequencing (scRNA-seq) has become the preferred method for determining the composition of complex tissues at the transcriptional level [12]. In contrast to traditional bulk RNA sequencing, scRNA-seq can reveal new cell types and rare subpopulations and can be used to explore genetic and functional heterogeneity at the single-cell level [13]. Some researchers have devoted themselves to providing a complete picture of the immune cellular composition and intercellular communication events that occur during normal pregnancy [14]. Moreover, scRNA-seq has been used in studies on reproductive diseases. On the basis of the evidence provided by scRNA-seq, the distributions of immune cell subsets have been proposed to differ dramatically between patients with recurrent pregnancy loss (RPL) and women with normal pregnancies [15]. Another study demonstrated the changes in some biological processes related to pregnancy, hormone secretion, and immunity in patients with preeclampsia (PE) [16]. In the present study, we collected decidual tissues from cases of voluntary abortion in the first trimester and purified human decidual immune cells. ScRNA-seq was employed to dissect decidual immune cells with or without *T. gondii* infection. The differentially expressed genes (DEGs) and Kyoto Encyclopedia of Genes and Genomes (KEGG) and gene ontology (GO) analysis results of dNK, dMφ, and dT cells and their subsets were horizontally compared. Transcription factor (TF) modules, their related target genes, and their predicted functions were also analyzed. Finally, a *T. gondii* infection-specific interaction network among dNK, dMφ, and dT cells and their ligand–receptor interactions, such as HLA-E–CD94:NKG2A and TNFSF13–TNFSF13B, was established. We discovered several immune cell subsets and novel molecules that might play a vital role in the occurrence of adverse pregnancy outcomes induced by *T. gondii* infection. Our results could provide convincing support for the future exploration of the molecular mechanism of *T. gondii* infection.

## Methods

### Human clinical sample collection

All voluntary abortions were obtained from Yantai Affiliated Hospital of Binzhou Medical University, Maternal and Child Health Care Hospital of Yantai Zhifu District, and Yantai Hospital of Traditional Chinese Medicine. Written informed consent was obtained from all the participants. Aborted tissues from healthy pregnant women in the first trimester (6–8 weeks) were collected in sterile saline, and decidual tissues were stored in Roswell Park Memorial Institute 1640 (RPMI-1640, HyClone, USA) medium containing 10% fetal bovine serum (FBS, Gibco, USA), 100 IU/mL penicillin, and 100 IU/mL streptomycin (Sigma-Aldrich, USA).

### Antibodies

The anti-VSIG4 antibody (Cat# bs-0479R, RRID: AB_10855328) and anti-TGF-beta antibody (Cat# bs-0086R, RRID: AB_10856457) were obtained from Bioss (China). The anti-GAPDH antibody (Cat# 10494-1-AP, RRID: AB_2263076) was obtained from Proteintech (China) and HRP-conjugated Affinipure goat anti-rabbit immunoglobulin G (IgG) (H + L) antibody (Cat# SA00001-2, RRID: AB_2722564) were obtained from Proteintech (China). The following human-specific mAbs were used: Pe-cy7-conjugated anti-CD14 (Cat# 25–0149-42, RRID: AB_1582276) and allophycocyanin APC-conjugated anti-VSIG4 (Cat# 17–5757-41, RRID: AB_2637396) were obtained from Thermo Fisher Scientific (USA). Fluorescein isothiocyanate (FITC)-conjugated anti-CD3 (Cat# 300406, RRID: AB_314060), PerCP/Cy5.5 conjugated anti-CD56 (Cat# 318322, RRID: AB_893389), and FITC-conjugated anti-line (Cat# 348801, RRID: AB_ 10612570) PerCP/Cy5.5 anti-HLA-DR (Cat# 307630, RRID: AB_893575) were obtained from Biolegend (USA).

### Preparation of single-cell suspensions

**Fig. 1:**
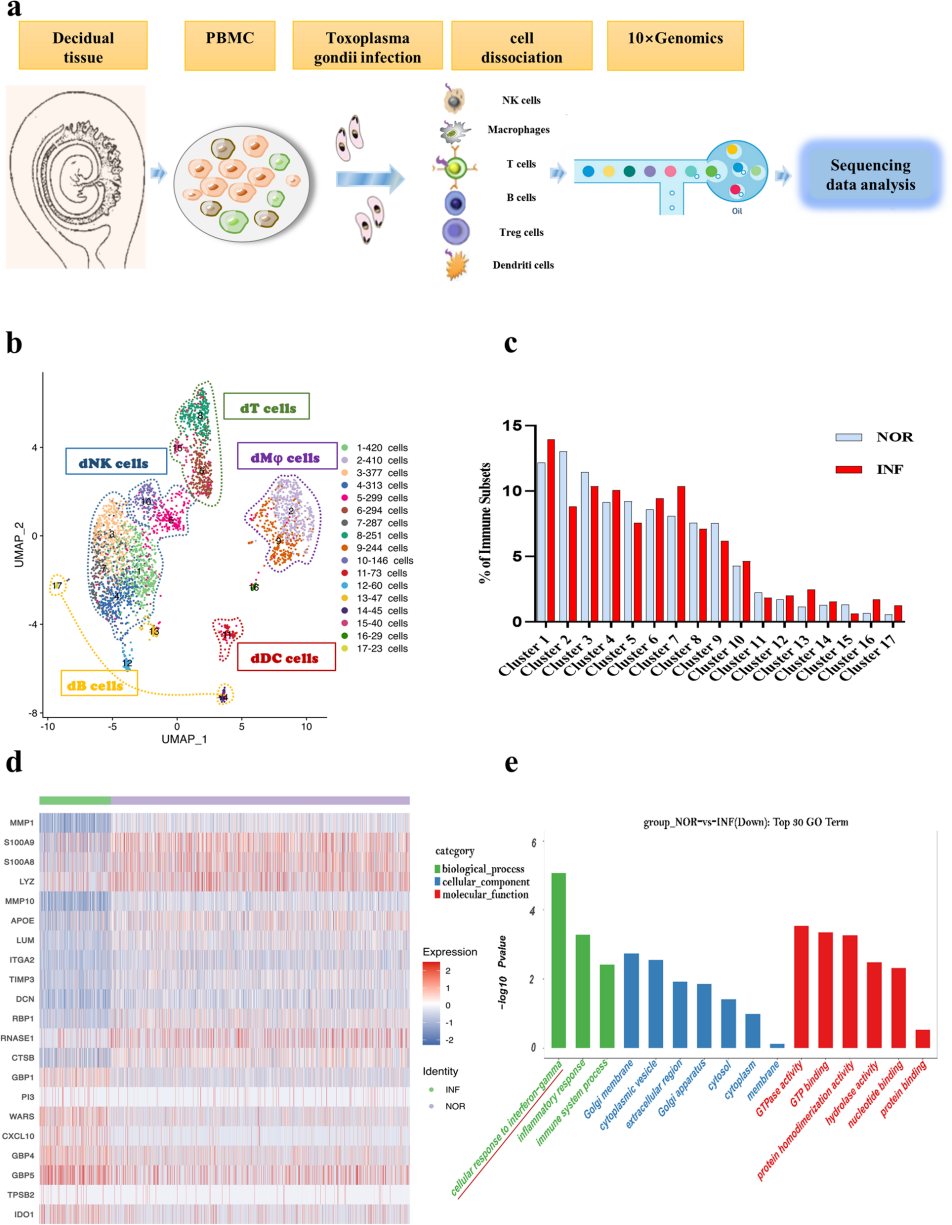
Overview of the single-cell landscape for human decidual immune cells and differential analysis between *T. gondii-*infected cells and uninfected cells.** a** The workflow of human decidual immune cells collection and purification for scRNA-seq.** b** The Uniform Manifold Approximation and Projection (UMAP) plot of 17 cluster decidual cells. **c** The proportions of difference of each cell cluster between *T. gondii*-infected cells and uninfected cells** d** Heatmap of the top ten differentially expressed genes of human decidual immune cells after *T. gondii* infection. **e** Gene ontology enrichment functional analysis of the upregulated DEGs after *T. gondii*-infection

The patient’s decidua was washed several times with cold phosphate buffered saline (PBS). Subsequently, the tissue was cut into small pieces and digested with 0.1% collagenase (Sigma, Germany) and 25 IU/mL DNase-I (Sigma, Germany) with shaking for 1 h at 37℃. Single-cell suspensions were obtained using a sterile mesh (48 µm). Mononuclear cells were collected from the PBMC after Ficoll density gradient centrifugation in lymphocyte isolation medium (TBD Science, China). Purified cells were equally divided into two groups: the normal group (NOR) and the infected group (INF). The INF group was treated with *T. gondii* (*T. gondii*:cell ratio of 1:5). The cells in the two groups were harvested after 24 h. The cell pellets were then resuspended in 1 mL of red blood cell lysis buffer and incubated for 10 min at 4 ℃. After red blood cell lysis, the samples were resuspended in PBS containing 0.04% BSA and fiber-filtered through a 40 µm cell strainer (VWR). The cell concentration and viability were determined via blood cell counting and trypan blue staining. The flowchart was shown in Fig. 1a.

### Single-cell RNA-seq data preprocessing

Database sequencing and data analysis were completed by Shanghai Oyi Biomedical Technology Co., Ltd. The raw data generated through high-throughput sequencing were in a fastq format sequence. The 10 × Genomics official software CellRanger (v. 5.0.0) was used to calculate the raw data quality and compare it with the reference genome. This software quantifies high-throughput single-cell transcriptome data by identifying barcode markers in sequences and the UMI (unique molecular identifier) markers of different mRNA molecules in each cell to obtain quality control statistics, such as high-quality cell number, gene median value, and sequencing saturation. On the basis of preliminary QC (Quality Control) with Cellranger, the Seurat [17] (v. 4.0.0) software package was used for the further QC processing of the data. Low-quality cells were filtered in accordance with the distribution of nUMI, nGene, and percent mito. The specific quality control scheme for DoubletFinder [18] (v. 2.0.2) software used to identify high-quality cells was as follows: cells with the number of genes greater than 200, the number of UMI greater than 1000, log10GenesPerUMI greater than 0.7, mitochondrial UMI ratio less than 5%, and proportion of red blood cell genes less than 5%.

### Dimensional reduction and cluster analysis

Dimensional reduction and cluster analysis were performed on hypervariable genes (HVGs, highly variable genes) using the FindVariableGenes function in the Seurat package. Principal component dimension reduction analysis was performed using the expression profiles of HVGs, and the results were visualized in two-dimensional space by UMAP (nonlinear dimension reduction).

### Identification of cluster marker genes and analysis of differential expression

Marker gene identification was performed using the FindAllMarkers function in the Seurat package, which found genes that were differentially upregulated in each cell subtype relative to other cell populations. These genes were considered the potential marker genes for each cell subtype. The resulting identified marker genes were visualized using the VlnPlot and FeaturePlot functions. Differentially expressed genes (DEGs) were identified using the Seurat package. *P*-value < 0.05 and foldchange > 1.2, or foldchange < 0.83 were set as the threshold for significant differential expression. GO and KEGG pathway enrichment analyses were performed on DEGs on the basis of hypergeometric distribution using R.

### Single-cell regulatory network inference and clustering (SCENIC) analysis

SCENIC analysis was run using the motif database for RcisTarget and GRNboost (SCENIC [19] version 1.1.2.2, which corresponds to RcisTarget 1.2.1 and AUCell 1.4.1) with default parameters. In detail, over-represented TF binding motifs were identified from a gene list with the RcisTarget package. The activity of each group of regulators (regulons) in each cell was scored using the AUCell package. The connection specificity index (CSI) of all regulons was calculated with the scFunctions (https://github.com/FloWuenne/scFunctions/) package.

### CellChat analysis

Cell communication analysis was performed using the CellChat [20] (v. 1.1.3) R package. A ligand or a receptor was defined as “expressed” in a particular cell type if 10% of the cells of that type had nonzero read counts for a ligand/receptor-encoding gene. Statistical significance was then assessed by randomly shuffling the cluster labels of all cells and repeating the above steps, which generated a null distribution for each ligand- receptor (LR) pair in each pairwise comparison between the two cell types. After running 1000 permutations, *P*-values were calculated using the normal distribution curve generated from the permuted LR pair interaction scores. Any two cell types wherein the ligand was expressed in the former cell type and the receptor was expressed in the latter were linked to define cell–cell communication networks. R packages Igraph and Circlize were used to display the obtained cell–cell communication networks.

### Reverse transcriptase polymerase chain reaction

The RNA of dNK cells or dMφ from the NOR and INF groups was isolated using Trizol and chloroform and then precipitated with isopropanol. cDNA was generated using a Thermo-Fisher High-Capacity cDNA Reverse Transcription Kit following the manufacturer’s instructions, and reverse transcriptase polymerase chain reaction (RT-PCR) was conducted using an RT-PCR synthesis kit (Qiagen, Germany) (Additional file 6: Table S1).

### Maintenance of *T. gondii* tachyzoites (RH strain)

*T. gondii* tachyzoites were cultured in human foreskin fibroblast (HFF) cells growing in RPMI-1640 medium (Dalian, China), 5% FBS (Gibco, USA), and 100 IU/mL penicillin/streptomycin (Sigma-Aldrich, USA). HFF cells were centrifuged at 800 rpm for 5 min after culture, and the clear supernatants were then centrifuged at 4000 rpm to purify tachyzoites. RPMI-1640 medium was used to resuspend the tachyzoites. The tachyzoites were then counted using a Neubauer chamber and cultured with new HFF cells. The experiments were conducted in BSL-2 laboratories. All the liquids, consumables, and labware contaminated with the parasites were collected, steeped immediately in disinfectant, and autoclaved.

### Isolation and purification of dNK, dMφ, dT

Decidual tissue was washed 5–7 times with cold PBS and then cut into 1–3 mm fragments with ophthalmic scissors. Subsequently, 1 mg/mL collagenase IV (Biofroxx, Germany) and 0.2 mg/mL DnaseI (Sigma‒Aldrich, St. Louis, USA) were added to digest the tissue in a biochemical incubator at 37 ℃ for 45 min. CD3^+^ dT cells and CD14^+^ dMφ were purified via immunomagnetic positive selection using a human CD3 positive selection kit and CD14 positive selection kit (Stem Cell Science) according to the manufacturer’s instructions with > 95% purity ensured for experiments, whereas CD3^−^CD56^+^ dNK were subjected to immunomagnetic negative selection by human NK cell isolation kit. Approximately 1 × 10^7^ purified decidual macrophages were allocated to the NOR, INF, and VSIG4-neutralized infected groups. CD14^+^ dMφ were incubated with 10 µg/mL anti-VSIG4 monoclonal antibody (mAb) in the VSIG4-neutralized infected group. After 1 h, *T. gondii* was added to the VSIG4-neutralized infected and INF groups at a ratio of 1:5 (*T. gondii*: cells). Study samples were cultured in RPMI medium supplemented with 10% FBS (FBS; Gibco, USA), 100 IU/mL streptomycin, and 100 IU/mL streptomycin (Sigma, USA) for 24 h at 37 °C in a humidified 5% CO_2_ incubator.

### Flow cytometry

Human decidual macrophages were incubated with the corresponding mAbs at 4℃ in the dark for 40 min and then washed with PBS once. Intracellular cytokines and enzymes were stained after cellular fixation and permeabilization. Analysis was performed with an FACSCanto™ II instrument (Becton Dickinson, USA).

### Western blot analysis

Equal amounts of protein were lysed with equal amounts of precooled lysis buffer containing 10 µL of the serine protease inhibitor phenylmethyl–sulfonyl fluoride per mL of buffer. After being incubated for 45 min on ice, the cell lysates were centrifuged at 12,000 × rpm at 4℃. The supernatant was collected, quantified using BCA (Bicinchoninic Acid) protein assay kits, and mixed with 5 × sodium dodecyl sulfate–polyacrylamide gel electrophoresis loading buffer. The sample was then boiled for 5 min. Subsequently, equal amounts of protein were separated through 12% sodium dodecyl sulfate–polyacrylamide gel electrophoresis. The proteins were transferred to polyvinylidene fluoride membranes (Millipore, Germany). The membranes were blocked at room temperature for 2.5 h in 5% nonfat dry milk in TBS-T. The membranes were incubated on a shaker overnight at 4 ℃ with rabbit anti-human VSIG4 (1:1,000, Bioss, China) and TGF-β (1:1,000, Bioss, China); GAPDH (1:40,000, Proteintech, China) was used as a loading control. The membranes were washed six times with TBS-T for 6 min each time and subsequently incubated with the appropriate secondary antibody for 2 h at room temperature. Immune complexes were visualized with an enhanced chemiluminescence detection kit (F. Hoffmann-La Roche, Ltd., Switzerland). Protein expression levels were determined using Image J software.

### Immunofluorescence analysis

Purified human CD14^+^dMφ cells from the NOR and INF groups were collected after 24 h of infection. All cells were washed and fixed for 40 min with 4% paraformaldehyde (PFA). After fixation in 4% PFA for 30 min, slides were washed with PBS and then blocked with goat serum for 1 h at room temperature. The cells were incubated overnight at 4℃ with anti-VSIG4 (1/500, Bioss) antibody and washed thrice with TBS-T. Following washing, the slides were incubated with appropriate concentrations of secondary antibodies for 1 h at 37 ℃. Dylight 549-rabbit anti-goat IgG (1/500, Bioss) was used as the secondary antibody. Finally, the slides were stained with a reagent containing Hoechst stain for 15 min and washed three times with PBS. The cells were placed in the confocal microscope chamber, and images were captured through confocal fluorescence microscopy (STELLARIS/5).

### Statistical analysis

Data were presented as the mean ± standard deviation. Statistical analyses were performed with GraphPad Prism 7 Statistics software package. Unpaired and paired *t*-tests were used to identify differences. Herein, *P* < 0.05 was regarded as significant and *P* < 0.01 was considered as highly statistically significant.

## Results

### Single-cell transcriptome profiling analysis of *T. gondii*-infected and uninfected human decidual immune cells

Aborted tissues from seven healthy pregnant women in their first trimester (6–8 weeks) were collected to investigate the potential immune molecules contributing to the adverse pregnancy outcomes induced by *T. gondii* infection. Mononuclear cells were purified and equally divided into the normal group (NOR) and *T. gondii*-infected (INF) group (Fig. 1a). A total of 34,864 cells (22,818 from normal samples and 12,046 from infected samples) were passed through stringent quality control filters before application in subsequent analysis. Among these cells, decidual immune cells were screened and re-clustered and referred to the particular marker PTPRC (Additional file 1: Fig. S1a and b). The results of analysis showed that a total of 6782 PTPRC-positive immune cells (1153 from normal samples and 5629 from infected samples) were obtained, and 10 × Genomics scRNA-seq was used for single-cell transcriptome profiling and for analyzing differences between two groups. A total of 17 cell clusters of immune cells were identified in accordance with their representative cell markers using the FindAllMarkers function in the Seurat package (Fig. 1b; Additional file 1: Fig. S1c). Clusters 1, 3–5, 7, and 10 were designated as the clusters of dNK cells with positive NCAM1 expression. Clusters 2 and 9 were classified as decidual macrophage cells on the basis of the CD14 marker. Clusters 6, 8, 12, 13, and 15 expressed the CD3E were categorized as decidual T cells. Clusters 14 and 17 expressing CD79A as a positive common marker were classified as decidual B cells, whereas the HLADR-positive cluster 11 was classified as decidual DC cells. The top ten highly expressed markers in each cluster were plotted and presented in the form of a heatmap in Additional file 2: Fig. S2. Interestingly, during *T. gondii* infection, the proportions of clusters 2 (dMφ), 3 (dNK), 5 (dNK), 8 (dCD8^+^T), 9 (dMφ), 11 (dDC), and 15 (dT) decreased, whereas those of clusters 1 (dNK), 4 (dNK), 6 (dCD4^+^T), 7 (dNK), 10 (dNK), 12 (dT), 13 (dT), 14 (dDC), 16 (dDC), and 17 (dB) increased (Fig. 1c). The notable DEGs were plotted in the form of a heatmap (Fig. 1d). The scRNA-seq data demonstrated that due to *T. gondii* infection, MMP1, S100A9, S100A8, LYZ, and MMP1 were significantly downregulated, whereas RBP1, RNASE1, CTSB, GBP1, and PI were upregulated in decidual immune cells. Subsequently, GO enrichment analysis revealed that the functions of the upregulated DEGs were relevant to the interferon (IFN)-γ signaling pathway (Fig. 1e).

### Analysis of changes in DEGs and functions of dNK cells and subsets after *T. gondii* infection

**Fig. 2:**
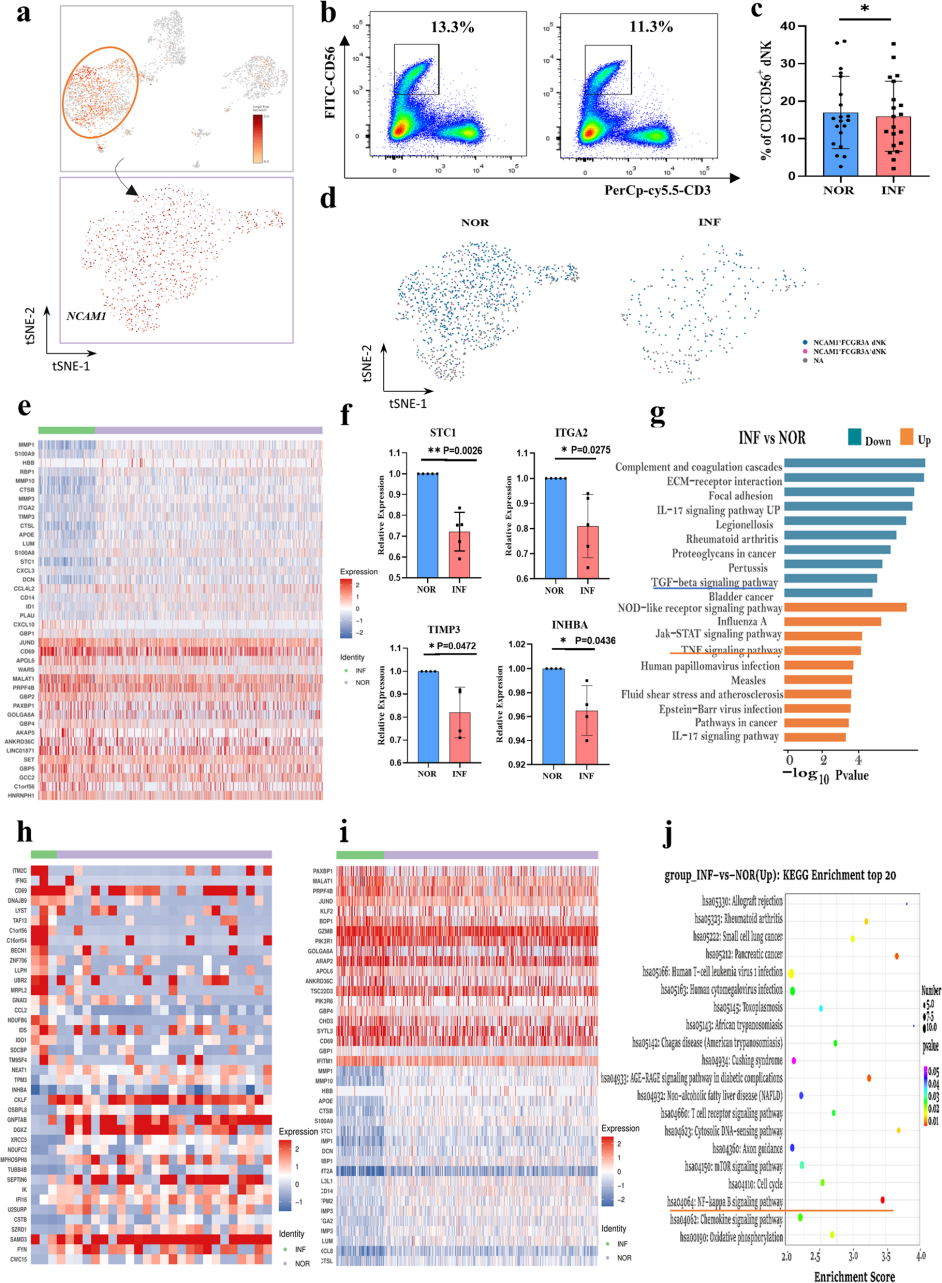
DEG analysis of dNK and dNK subsets after *T. gondii* infection. **a** The circled of decidual NK cells from total immune cells. **b**, **c** The percentage changes of human decidual NK cells with flow cytometry analysis (*n* = 20 per group). Data are presented as mean ± SD, *P < 0.05, ** *P* < 0.01, by paired *t*-test, NOR, normal group; INF, RH-infected group. **d** The t-SNE map of NCAM1^+^FCGR3A^+^ dNK and NCAM1^+^FCGR3A^−^ dNK in infected cells and uninfected cells. **e** Heatmap of top 20 DEGs between *T. gondii*-infected and uninfected dNK cells. **f** RT-PCR analysis of partial top 20 differential genes (*n* = 4–9 per group). Data are presented as the mean ± SD, *P < 0.05, ** *P* < 0.01, by paired *t*-test, NOR, normal group; INF, RH-infected group. **g** The histogram of top DEGs in dNK cells by KEGG analysis. **h** Heatmap of the DEGs in NCAM1^+^FCGR3A^+^dNK.** i** Heatmap of the differential gene expression in NCAM1^+^FCGR3A^−^dNK. **j** KEGG analysis of upregulated genes in NCAM1^+^FCGR3A^+^dNK

dNK cells were reclustered from immune cells positive for the classical marker gene NCAM1 to further comprehend their heterogeneity during *T. gondii* infection (Fig. 2a). The result of flow cytometry showed that the number of dNK cells decreased after *T. gondii* infection (Fig. 2b, c). The expression profiles of the dNK cell subsets (NCAM1^+^FCGR3A^−^ and NCAM1^+^FCGR3A^+^) are presented with t-SNE plots in Fig. 2d. Among the 279 DEGs, 97 were upregulated and 182 were downregulated (P < 0.05, foldchange > 1.2, or foldchange < 0.83) compared with those in the NOR group. The top 20 DEGs in dNK cells are shown in the form of a heatmap (Fig. 2e). We focused on the genes (STC1, INHBA, ITGA2, and TIMP3) that were significantly downregulated after *T. gondii* infection as identified through scRNA-seq analysis for further quantitative revers RT-PCR validation (Fig. 2f). GO enrichment analysis revealed that the function of the enriched upregulated genes was mostly related to the TNF signaling pathway, whereas that of the enriched downregulated genes was mostly related to the TGF-β signaling pathway (Fig. 2g). DEGs in the dNK subsets (NCAM1^+^FCGR3A^−^dNK and NCAM1^+^FCGR3A^+^dNK) were further analyzed and shown in Fig. 2i and h. MMP1, STC1, TIMP1, TIMP3, and ITGA2 were significantly downregulated, whereas GZMB, JUND, GBP4, and IFITM1 were upregulated in NCAM1^+^FCGR3A^−^ dNK cells after *T. gondii* infection (Fig. 2i). The significantly downregulated genes (NEAT1, TPM3, ZNHBA, CKLF, and OSBPL8) and the upregulated genes (ITMZC, IFNG, CD69, DNAJB9, and LYST) in NCAM1^+^FCGR3A^+^dNK cells after infection were also analyzed (Fig. 2h). KEGG enrichment analysis revealed that the functions of some enriched upregulated genes in NCAM1^+^FCGR3A^+^dNK cells were related to immune response, specifically to the NF-κβ signaling pathway (Fig. 2j).

### SCENIC analysis of the transcription factor heterogeneity of dNK cells after *T. gondii* infection

**Fig. 3:**
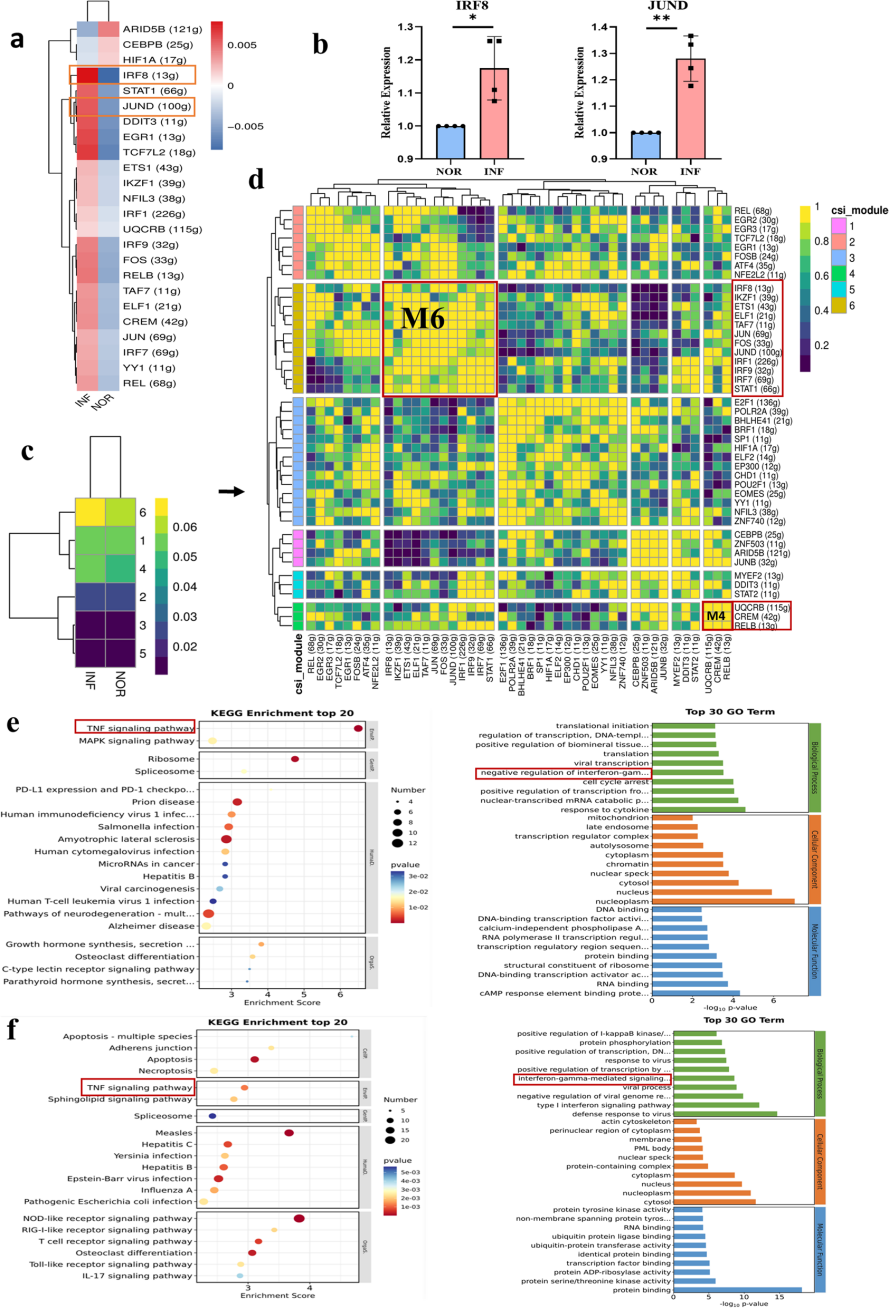
Transcription factor heterogeneity SCENIC analysis of dNK after *T. gondii* infection. **a** SCENIC analysis of the decidual NK cells (NOR versus INF). **b** The RT-PCR validation of IRF8 and JUND (*n* = 4 per group).Data are presented as mean ± SD, *P < 0.05, ** *P* < 0.01, by paired *t*-test, NOR, normal group; INF, RH-infected group. **c** The identification of difference regulons between infected and uninfected dNK cells. **d** The enrichment of difference regulons by CSI. **e** The KEGG and GO analysis of 12 regulons in M6 module. **f** The KEGG and GO analysis of three regulons in M4 module

The changes in TFs in dNK cells after *T. gondii* infection were analyzed with Single Cell Regulatory Network Inference and Clustering (SCENIC). The regulon activity scores (RAS) showed that IRF8 and JUND were the most significantly upregulated TFs, and they regulated the expression levels of 12 and 99 target genes, respectively (Fig. 3a). RT-PCR was performed on the TFs IRF8 and JUND to validate the results of SCENIC analysis (Fig. 3b). Each TF and its potential direct target genes were considered as regulons. In accordance with connection specificity index (CSI) values, the regulons that differed between infected and uninfected dNK cells were identified and presented with different colors in Fig. 3c. Regulons were organized into six major modules (M1–M6) in a regulon CSI matrix. M6 and M4 were identified as active modules in infected and uninfected dNK cells (Fig. 3d). M6 contained 12 regulons, including ELF1 (21 g), ETS1 (43 g), FOS (33 g), IKZF1 (39 g), IRF1 (226 g), IRF7 (69 g), IRF8 (13 g), IRF9 (32 g), JUN (69 g), JUND (100 g), STAT1 (66 g), and TAF7 (11 g). M4 contained three regulons, namely, CREM (42 g), RELB (13 g), and UQCRB (115 g). Interestingly, the results of KEGG and GO analyses suggested that during *T. gondii* infection, the function of the target genes regulated by M6 and M4 TFs might be involved in the TNF and IFN-γ signaling pathways (Fig. 3e, f).

### Analysis of DEGs in dMφ after *T. gondii* infection

**Fig. 4:**
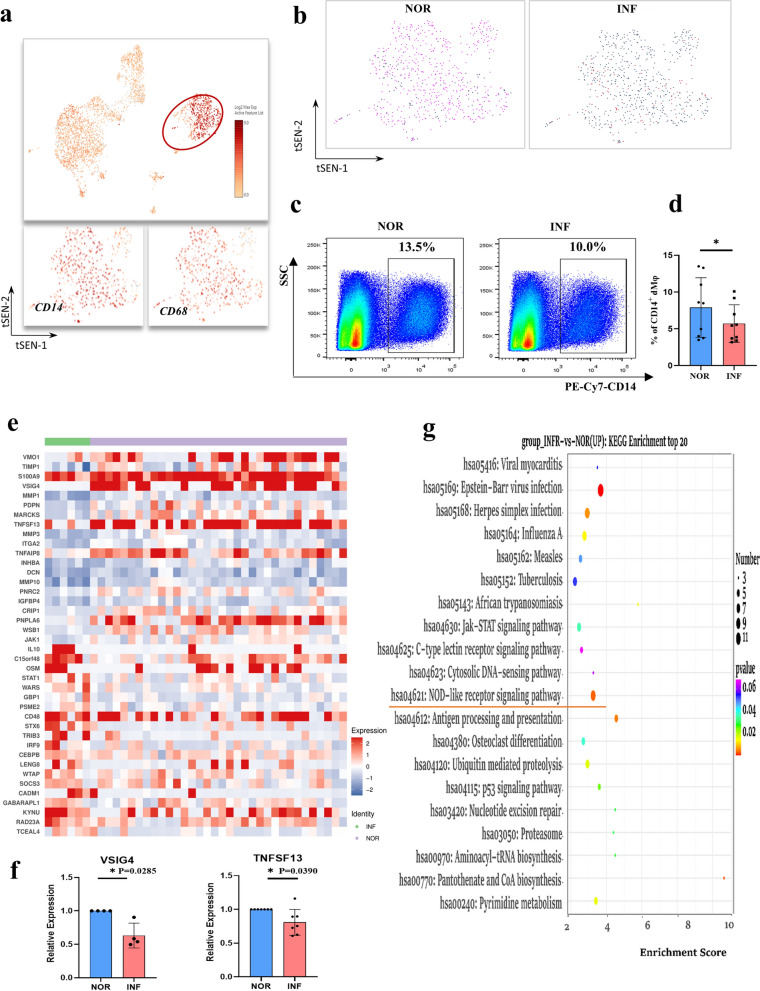
DEG analysis of dMφ after *T. gondii* infection. **a** The circled of dMφ from total immune cells. **b** The t-SNE map of infected dMφ and normal dMφ. **c**, **d** The percentage changes of dMφ in infected and normal cells with flow cytometry analysis(*n* = 9). Data are presented as mean ± SD, *P < 0.05, ** *P* < 0.01, by paired *t*-test, NOR, normal group; INF, RH-infected group. **e** Heatmap of top 20 differential gene expression between infected and normal dMφ. **f** The relative expression of VSIG4 and TNFSF13B genes by RT-PCR (*n* = 4–7 per group). Data are presented as mean ± SD, *P < 0.05, ** *P* < 0.01, by paired *t*-test, NOR, normal group; INF, RH-infected group. **g** KEGG enrichment analysis of significantly upregulated genes in dMφ cells with *T. gondii* infection

CD14- or CD68-positive dMφ cells were reclustered to further clarify the heterogeneity of dMφ during *T. gondii* infection (Fig. 4a). The t-SNE map showing the proportion of dMφ cells in infected and uninfected cells (Fig. 4b). Flow cytometry was used to analyze the changes in the number of dMφ cells after *T. gondii* infection. The results showed that the number of dMφ cells decreased after *T. gondii* infection (Fig. 4c and d). A total of 312 genes were differentially expressed (*P* < 0.05, foldchange > 1.2, or foldchange < 0.83) after *T. gondii* infection. They included 256 and 56 upregulated and downregulated genes, respectively. The top 20 DEGs in dMφ induced by infection are shown in the form of a heatmap (Fig. 4e). The RT-PCR verification results of the significantly downregulated genes VSIG4 and TNFSF13B induced by *T. gondii* infection were consistent with the scRNA-seq analysis results (Fig. 4f). Furthermore, the function of the upregulated genes was analyzed through KEGG. KEGG results demonstrated that the upregulated genes may be involved in some signaling pathways related to the NOD-like receptor (Fig. 4g).

### Analysis of DEGs in dMφ subsets after *T. gondii* infection

**Fig. 5:**
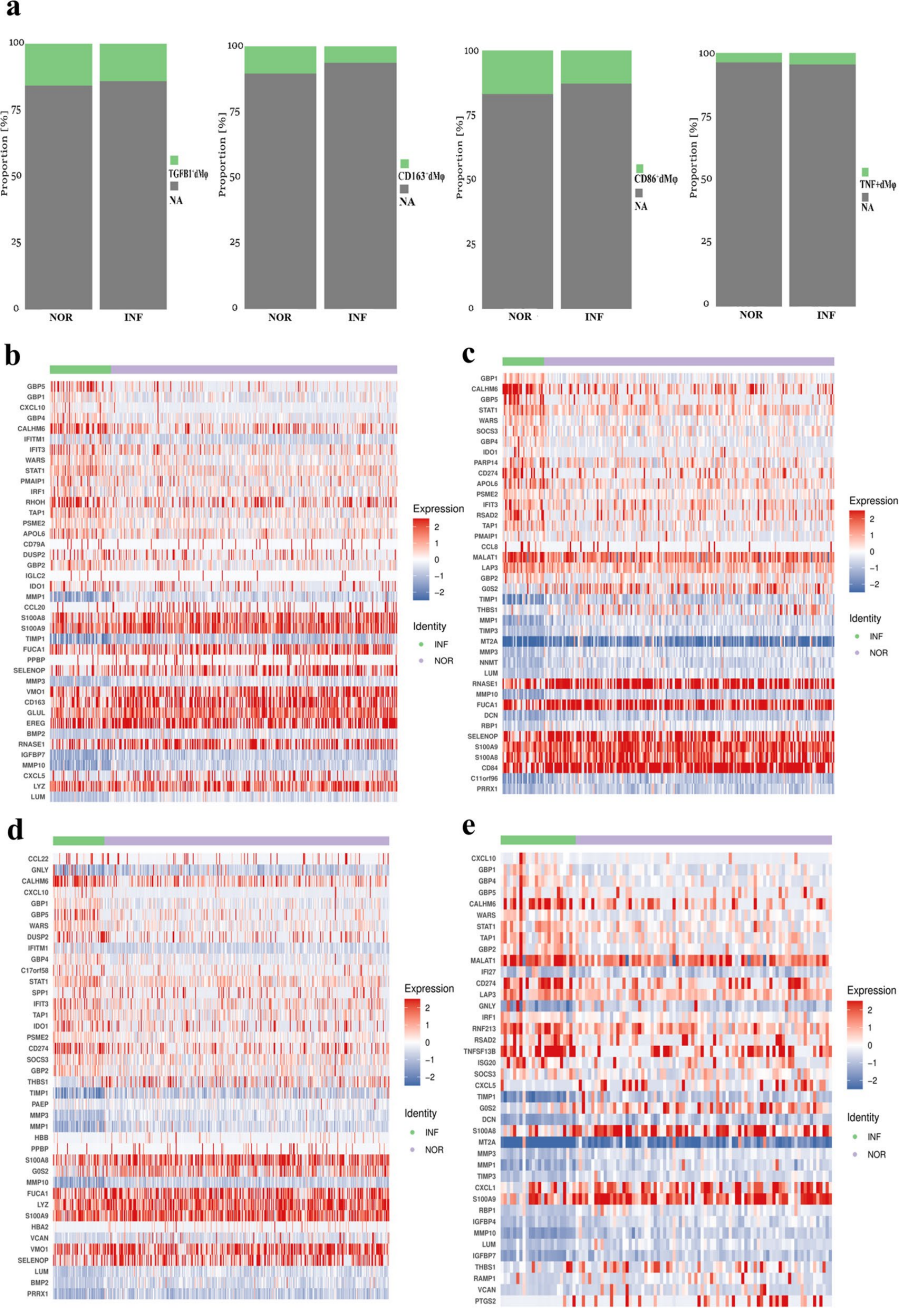
DEG analysis of dMφ subsets after *T. gondii* infection. **a** The t-SNE map of TGFB1^+^ dMφ, CD163^+^ dMφ, CD86^+^ dMφ and TNF^+^ dMφ. **b** Heatmap of the differential genes expression in TGFB1^+^ dMφ. **c** Heatmap of the differential gene expression in CD163^+^ dMφ. **d** Heatmap of the differential genes expression in CD86^+^ dMφ. **e** Heatmap of the differential gene expression in TNF^+^ dMφ

TNF^+^ dMφ, CD86^+^ dMφ, CD163^+^ dMφ, and TGFβ^+^ dMφ subsets were presented in the form of t-SNE plots in Fig. 5a to observe the differences induced by *T. gondii* infection in dMφ subsets. The marker genes of four subsets were presented in Additional file 3: Fig. S3. Moreover, the DEGs in the dMφ subsets are shown in Fig. 5b–e. In TGFβ1^+^dMφ cells, MMP1, CCL20, S100A8, S100A9, and TIMP1 were significantly downregulated, whereas GBP5, GBP1, CXCL10, GBP4, and CALHM6 were upregulated. Meanwhile, in CD163^+^dMφ cells, GOS2, TIMP1, THBS1, MMP1, and TIMP3 were downregulated, whereas GBP1, CALHM6, GBP5, STAT1, CALHM6, and WARS were upregulated. In CD86^+^dMφ cells, THBS1, TIMP1, PAEP, MMP3, and MMP were significantly downregulated, whereas CCL22, GNLY, CALHM6, CXCL10, GBP1, and GBP5 were upregulated. In TNF^+^dMφ cells after *T. gondii* infection, MMP1 TIMP1, MARCKS, DCN, and TIMP3 were downregulated, whereas GBP1, CALHM6, GBP4, SOCS3, and GBP5 WARS were upregulated.

### SCENIC analysis of TF heterogeneity in dMφ after *T. gondii* infection

**Fig. 6:**
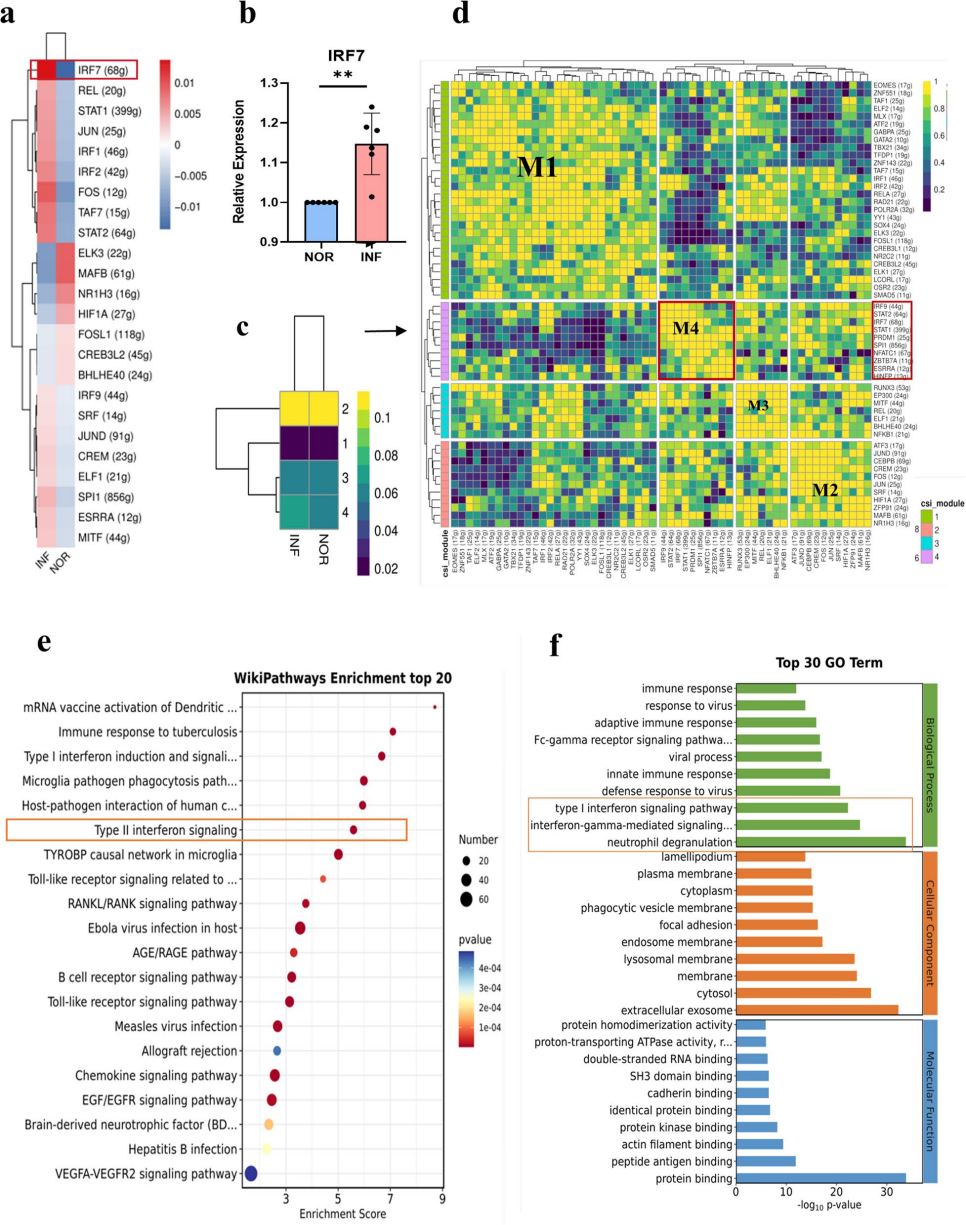
Transcription factor heterogeneity SCENIC analysis of dMφ after *T. gondii* infection. **a** RAS activity heatmap of dMφ (NOR versus INF). **b** RT-PCR validation of IRF7 (*n* = 6 per group). Data are presented as mean ± SD, *P < 0.05, ** *P* < 0.01, by paired *t*-test, NOR, normal group; INF, RH-infected group. **c**, **d** Four distinct modules classified from regulons by CSI. **e**, **f** The WikiPathways and GO analysis of 10 regulons in M4

SCENIC analysis was used to infer the RAS of dMφ with *T. gondii* infection (Fig. 6a). RAS showed that IRF7, which could regulate the expression of 67 target genes, was the most significantly upregulated TF. Subsequently, RT-PCR was performed to validate the result of the IRF7 SCENIC analysis (Fig. 6b). Each TF and its potential direct target genes were considered regulators (regulons). The regulons that differed between infected and uninfected dMφ cells were assigned different colors in accordance with their CSI values (Fig. 6c). Total regulons were organized into four major modules (M1–M4) in accordance with the CSI matrix. M4 was identified as the most active module in infected and uninfected dMφ cells. It contained ESRRA (12 g), HINFP (13 g), IRF7 (68 g), IRF9 (44 g), NFATC1 (67 g), PRDM1 (25 g), SPI1 (856 g), STAT1 (399 g), STAT2 (64 g), and ZBTB7A (11 g) regulons (Fig. 6d). Interestingly, the function of M4 TF-related target genes were analyzed with WikiPathways and GO. GO analysis results indicated that M4 TF-related target genes might be involved in IFN I and II signaling pathways and participate in neutrophil degranulation during *T. gondii* infection (Fig. 6e, f).

### Functional molecule expression in dMφ regulated by VSIG4 downregulation after *T. gondii* infection

**Fig. 7:**
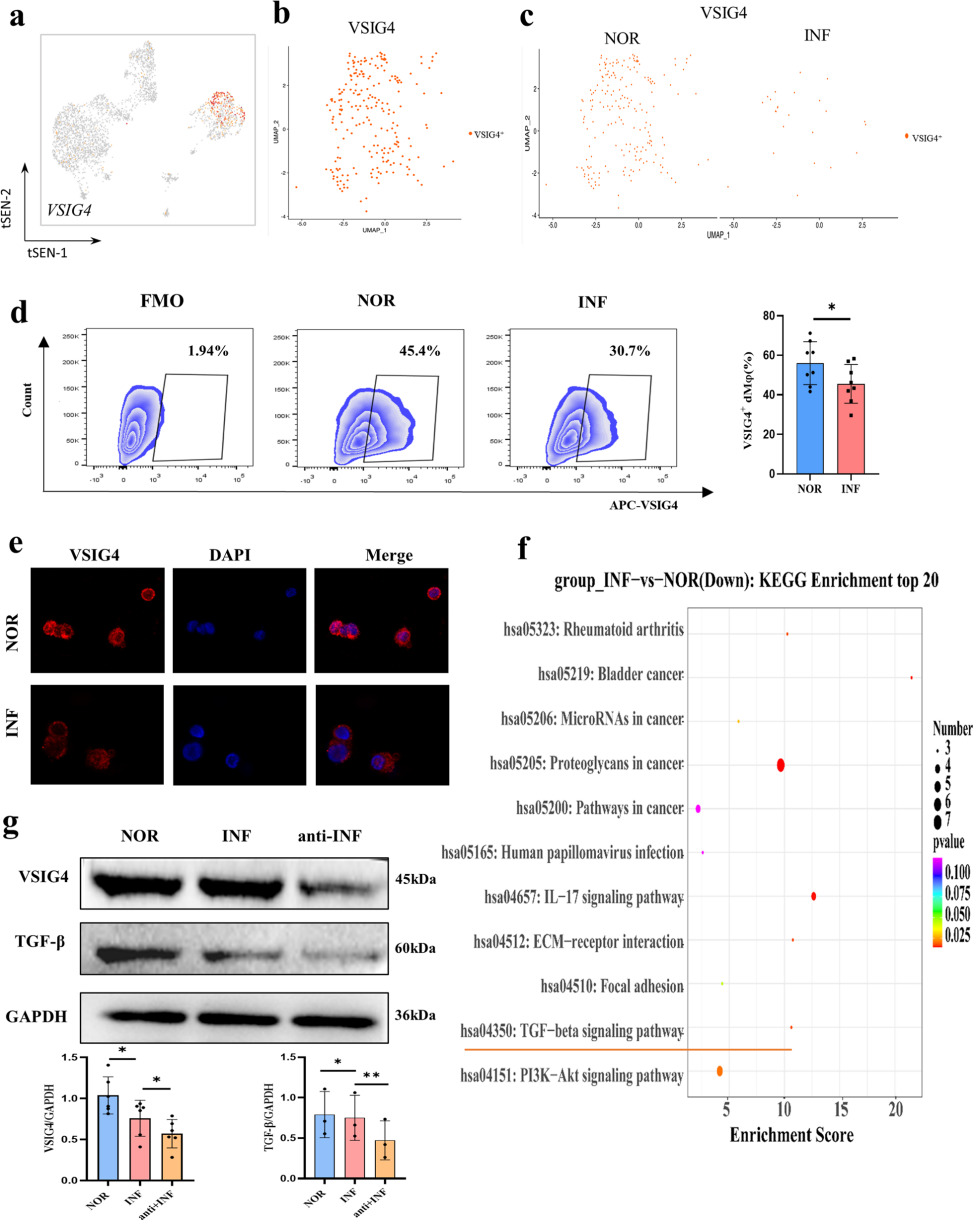
The downregulation of VSIG4 could regulate the expression of TGF-beta. **a** The t-SNE map of VSIG4^+^ decidual immune cells. **b** The t-SNE map of VSIG4^+^ dMφ. **c** The VSIG4^+^dMφ cells t-SNE map of infected dMφ and normal dMφ. **d** The mean fluorescence intensity of VSIG4 in normal and infected dMφ cells with immunofluorescence analysis (*n* = 8 per group). Data are presented as mean ± SD, *P < 0.05, ** *P* < 0.01, by paired *t*-test, NOR, normal group; INF, RH-infected group. **e** The VSIG4 expression on human dMφ and the difference between infected and normal dMφ cells with flow cytometry analysis (*n* = 8 per group). Data are presented as mean ± SD, *P < 0.05, ** *P* < 0.01, by paired *t*-test, NOR, normal group; INF, RH-infected group. **f** The top downregulated genes in VSIG4^+^dMφ after infection with KEGG analysis. **g** The changes of VSIG4 and TGF-β expression level in purified dMφ from three groups (normal, infected, and VSIG4-neutralized plus infected groups) with western blotting analysis. Data are presented as mean ± SD, *P < 0.05, ** *P* < 0.01, by paired *t*-test, NOR, normal group; INF, RH-infected group; anti + INF, VSIG4-neutralized plus infected groups

Study demonstrated that VSIG4 acted as a co-inhibitory ligand and could negatively regulate T cell proliferation and cytokine production [21]. VSIG4 was restrictedly expressed on macrophages [22]. In this study, scRNA-seq analysis showed that VSIG4 was highly expressed on human dMφ cells (Fig. 7a and b) but not on dNK cells and dDC (Additional file 4: Fig. S4). The number of VSIG4^+^dMφ cells decreased after *T. gondii* infection (Fig. 7c). The RT-PCR validation results were consistent with scRNA-seq data (Fig. 4f). Immunofluorescence analysis and flow cytometry were used to explore the role of VSIG4 in dMφ during *T. gondii* infection. The expression level of VSIG4 significantly decreased in human decidual macrophages after *T. gondii* infection (Fig. 7d and e). Interestingly, the result of KEGG pathway enrichment analysis showed that the downregulated genes in VSIG4^+^dMφ after *T. gondii* infection were associated with several signaling pathways, including the TGF-β signaling pathway (Fig. 7f). To further explore the effect of VSIG4 downregulation after infection on the functional molecules of dMφ, functional molecules TGF-β were analyzed by western blotting (Fig. 7g). The production of the functional molecule TGF-β decreased in infected dMφ cells and reduced after treatment with the VSIG4-neutralized antibody.

### Analysis of DEGs in dT after *T. gondii* infection

**Fig. 8:**
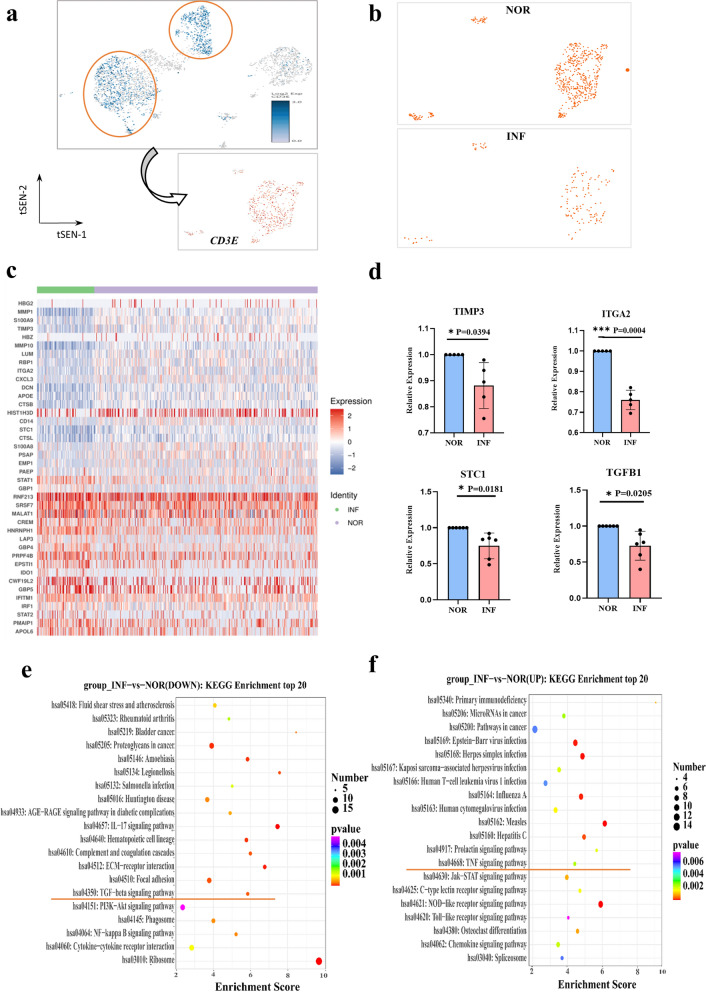
DEG analysis of dT after *T. gondii* infection. **a** The circled of decidual T cells from total immune cells.** b** The CD3E^+^dT cells t-SNE map of infected dT and normal dT cells. **c** Heatmap of top 20 differential gene expression between infected and uninfected dT cells. **d** RT-PCR analysis of partial top differential genes (*n* = 4–7 per group). Data are presented as mean ± SD, *P < 0.05, ** *P* < 0.01, by paired *t*-test, NOR, normal group; INF, RH-infected group. **e**, **f** KEGG enrichment analysis of significantly upregulated and downregulated genes in dT cells after *T. gondii* infection

dT cells were re-clustered from CD3E-positive cells to further comprehend the heterogeneity of decidual T cells during *T. gondii* infection (Fig. 8a). The scRNA-seq data showed that the number of decidual T cells decreased after *T. gondii* infection (Fig. 8b and c). A heatmap was used to reveal the top 20 DEGs in decidual T cells (Fig. 8d). Among the 380 DEGs after *T. gondii* infection, 205 were upregulated, whereas 175 were downregulated (*P* < 0.05, foldchange > 1.2, or foldchange < 0.83). The genes (TIMP3, STC1, ITGA2, and TGFΒ1) showing significant downregulation induced by *T. gondii* infection identified through scRNA-seq analysis were further validated with RT-PCR (Fig. 8d). Additionally, the downregulated and upregulated genes were subjected to KEGG enrichment analysis. Interestingly, the function of the downregulated genes was specifically associated with the TGF-β signaling pathway (Fig. 8e), whereas that of the upregulated genes was mainly related to the TNF signaling pathway (Fig. 8f).

### SCENIC analysis of the TF heterogeneity of dT after *T. gondii* infection

**Fig. 9:**
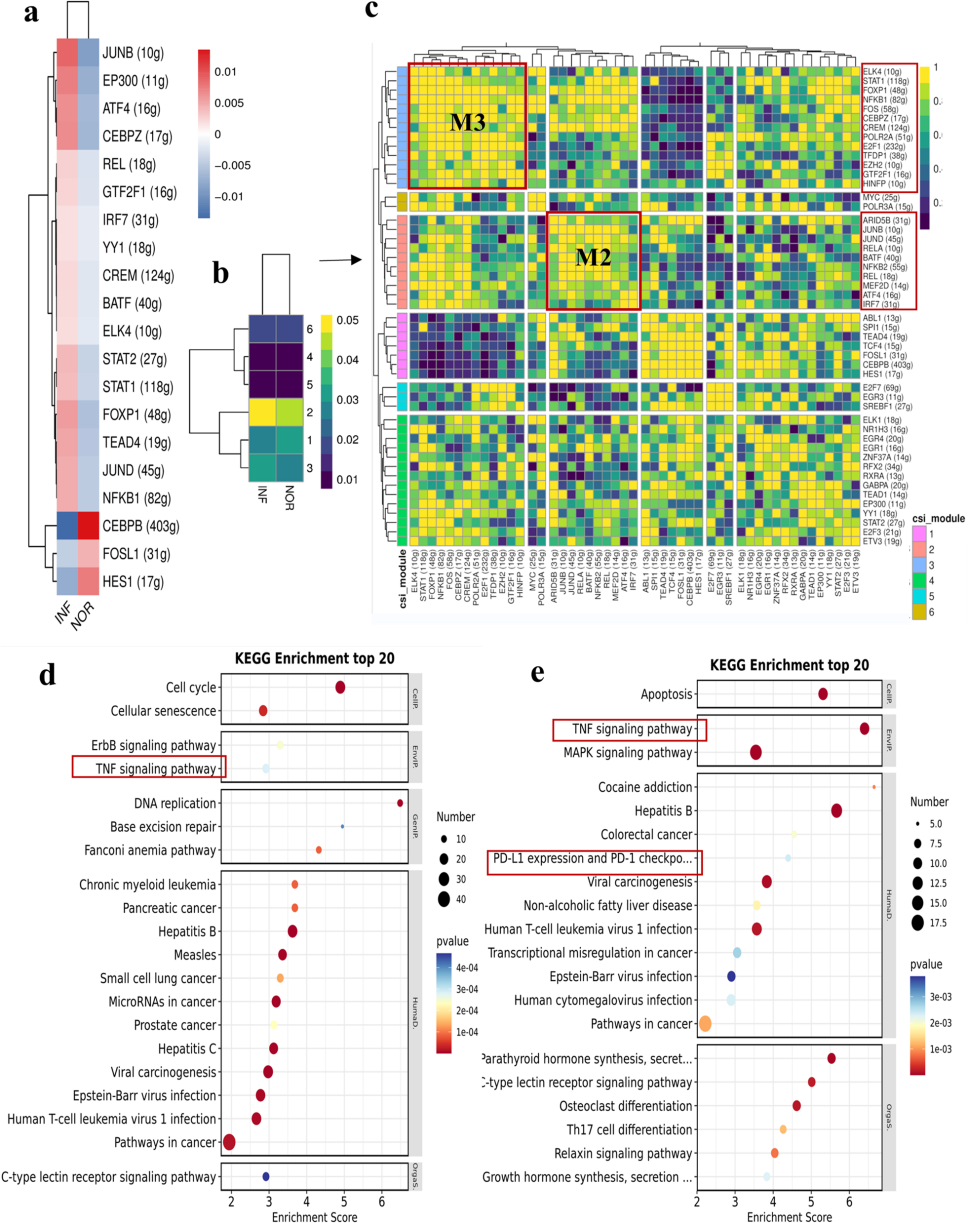
Transcription factor heterogeneity SCENIC analysis of dT after *T. gondii* infection. **a** RAS activity heatmap of dT (NOR versus INF).** b**, **c** Six distinct modules classified from regulons by CSI. **d** KEGG analysis of 13 regulons in M3 module. **e** KEGG analysis of ten regulons in M2 module

To estimate the regulon activity score (RAS) for dT with *T. gondii* infection, SCENIC analysis was used (Fig. 9a). According to connection specificity index (CSI) values, the different regulons between infected and uninfected dT cells were identified with different colors (Fig. 9b). Regulons were organized into six major modules (M1–M6) using the regulon CSI matrix (Fig. 9c). M3 and M2 were identified as active modules in infected and uninfected dT cells. M3 contained 13 regulons, including CEBPZ (17 g), CREM (124 g), E2F1 (232 g), ELK4 (10 g), EZH2 (10 g), FOS (58 g), FOXP1 (48 g), GTF2F1 (16 g), HINFP (10 g), NFKB1 (82 g), POLR2A (51 g), STAT1 (118 g), and TFDP1(38 g). M2 contained 10 regulons, namely, ARID5B (31 g), ATF4 (16 g), BATF (40 g), IRF7 (31 g), JUNB (10 g), JUND (45 g), MEF2D (14 g), NFKB2 (55 g), REL (18 g), and RELA (10). KEGG analysis revealed that the target genes regulated by M3 and M2 TFs (Fig. 9d and e) might be involved in the TNF-mediated signaling pathway during *T. gondii* infection. The regulon of M2 also participated in the PD1–PDL1-related signaling pathway induced by *T. gondii* infection.

### Changes in cell–cell communication networks in human decidual immune cells after *T. gondii* infection

**Fig. 10:**
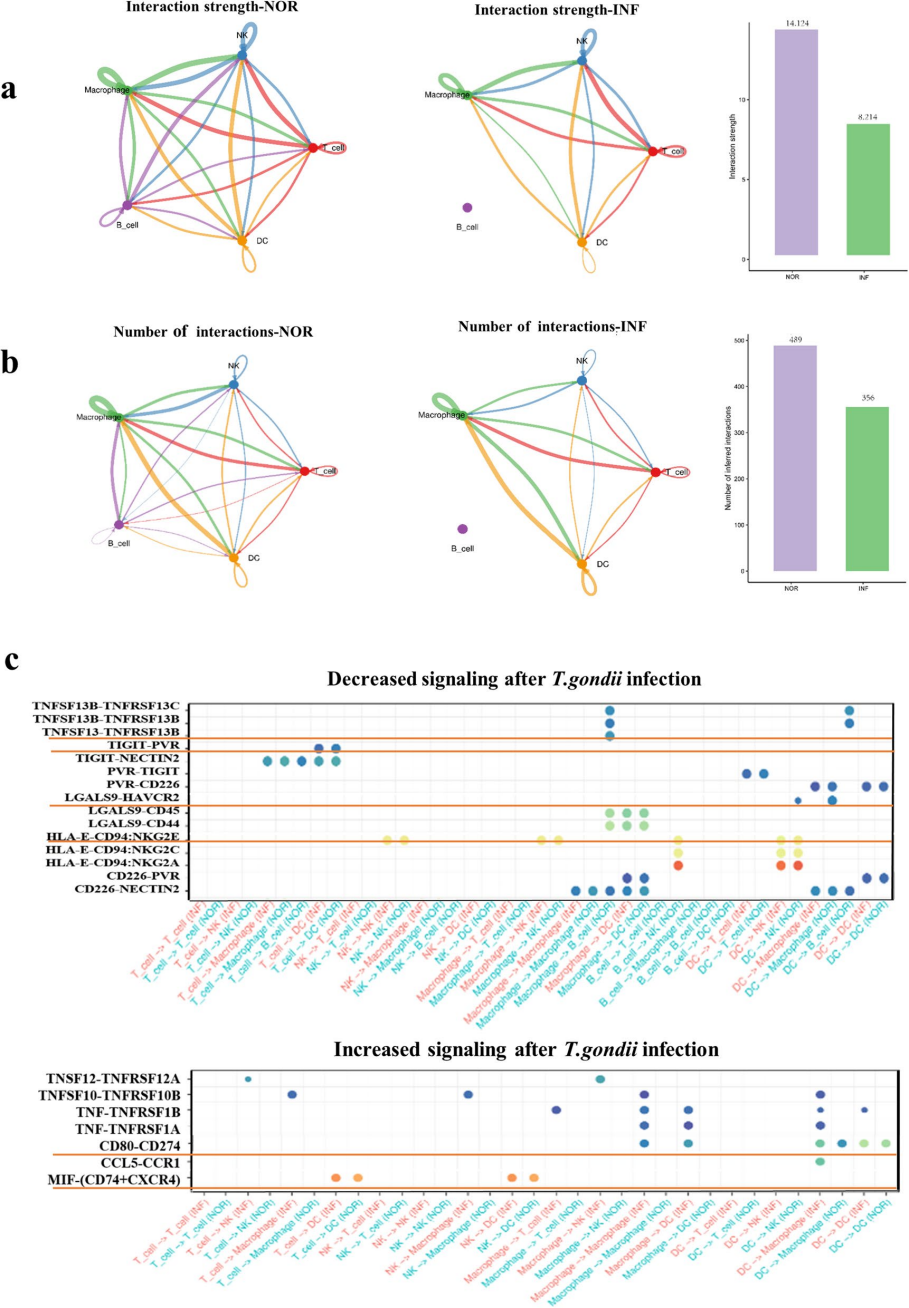
The changes of intercellular communications among different immune cells after *T. gondii* infection. **a** The strength map of the receptor-ligand interaction among five kinds of decidual immune cells in the infected and uninfected groups. **b** The number map of the receptor-ligand interaction among five kinds of decidual immune cells in the infected and uninfected groups. **c** The changes of ligand-receptor signal connections among five kinds of decidual immune cells after *T. gondii* infection

The cooperation and coordination of decidual immune cells with each other are critically involved in maintaining the balance of maternal–fetal immune tolerance. To observe the communication changes among the five types of human decidual immune cells, the intercellular receptor–ligand pairs and molecular interactions of immune cells were analyzed using the CellPhoneDB algorithm (NOR versus INF). Interestingly, the results showed that the strength and number of the receptor–ligand interactions among the decidual immune cells significantly weakened after *T. gondii* infection (Fig. 10a and b). Specifically, our analysis further identified the diverse receptor–ligand interplays of the five types of decidual immune cells in the INF and uninfected groups (Fig. 10c). Notably, some inhibitory interactions, such as TNFSF13–TNFSF13B, TIGIT–PVR, TIGIT–NECTIN2, HLA-E:CD94–NKG2, and LGALS9–HAVCR2, were weakened, whereas some active interactions, such as MIF–(CD74^+^CXCR4) and CD80–CD274, enhanced after *T. gondii* infection. Furthermore, the signaling pathway network of IL-2 and CD80 were enhanced, whereas the signaling pathway of TIGIT were weakened due to *T. gondii* infection (Additional file 5: Fig. S5a and b).

## Discussion

As a revolutionary technology, scRNA-seq has become the preferred method for determining the composition of complex tissues at the transcriptional level [13]. Some researchers have devoted themselves to providing a comprehensive picture of the immune cellular composition and intercellular communication events during normal pregnancy [14]. Moreover, on the basis of the evidence provided by scRNA-seq, the occurrence of adverse pregnancy outcomes, such as recurrent pregnancy loss and preeclampsia, have been proposed to be associated with the dysfunction of decidual immune cells [15, 16]. *T. gondii* infection could disrupt the immune microenvironment of the maternal–fetal interface contributing to the occurrence of adverse pregnancy outcomes, especially during the first trimester [2]. However, the mechanism underlying the disruption of maternal–fetal immune tolerance is complicated. Numerous unknown immune molecules in decidual immune cells may participate in the above process and contribute to adverse pregnancy outcomes during *T. gondii* infection. ScRNA-seq can be used to identify the unknown molecules that might play a vital role in the occurrence of abnormal pregnancy outcomes during *T. gondii* infection. In the present study, we subjected infected human decidual immune cells to scRNA-seq. Our results showed that the proportion of 17 decidual immune cell clusters changed after *T. gondii* infection. FindAllMarkers identified six clusters of dNK, two clusters of dMφ, five clusters of dT, two clusters of dB cell, and one cluster of dDC in accordance with the representative cell markers. Interestingly, the proportion of clusters 2 (dMφ), 3 (dNK), 5 (dNK), 8 (dCD8^+^ T), 9 (dMφ), 11 (dDC), and 15 (dT) decreased, whereas those of clusters 1 (dNK), 4 (dNK), 6 (dCD4^+^ T), 7 (dNK), 10 (dNK), 12 (dT), 13 (dT), 14 (dDC), 16 (dDC), and 17 (dB) increased during *T. gondii* infection. Our results demonstrated that the proportion of several primary decidual immune cells and subsets changed due to *T. gondii* infection, which eventually lead to the imbalance of immune microenvironment at the maternal–fetal interface. We also found that genes MMP1, S100A9, S100A8, LYZ, and MMP1 were significantly downregulated, while those genes (RBP1, RNASE1, CTSB, GBP1, and PI) were upregulated in decidual immune cells induced by *T. gondii* infection. These results revealed that the above different genes might provide new clues for understanding the mechanisms and therapeutic strategies for adverse pregnancy outcomes caused by *T. gondii* infection. Moreover, GO enrichment analysis demonstrated that the functions of those differentially upregulated genes were relevant to the interferon-gamma signal pathway. This result indicated that the killing function of decidual immune cells might be enhanced during *T. gondii* infection, thereby contributing to the occurrence of adverse pregnancy outcomes.

Studies have shown that dNK cells are the largest population (70%) of decidual leukocytes during the first trimester and execute multiple functions to maintain immune homeostasis during pregnancy [23]. A previous study has shown that *T. gondii* infection can change the expression levels of numerous inhibitory molecules (Tim-3 and 2B4), resulting in the dysfunction of dNK [6, 24]. However, the number of key molecules in dNK cells that changed and played important roles in the occurrence of abnormal pregnancy outcomes during *T. gondii* infection remains unclear. In the present study, we analyzed the heterogeneity of dNK cells during *T. gondii* infection through scRNA-seq. Our data showed that the number of dNK cells decreased after *T. gondii* infection and were further validated with flow cytometry. This result demonstrated that the function maintaining immune homeostasis during pregnancy was weakened due to a decline in the number of dNK cells. The analysis of DEGs in dNK cells showed that there were 97 genes up-regulated and 182 genes downregulated among the 279 DEGs induced by *T. gondii* infection. Our results revealed that numerous unknown genes changed during *T. gondii* infection, which provided several potential key molecules for further exploration to elucidate the mechanism of adverse pregnancy outcomes. We focused on the genes (STC1, INHBA, ITGA2, and TIMP3) that scRNA-seq analysis identified as significantly downregulated after *T. gondii* infection and further subjected them to RT-PCR validation. Evidence proposed that the dysregulation of STC1, TIMP3, ITGA2, and INHBA is associated with the development of severe reproductive diseases [25–29]. Therefore, our results indicated that the change in the expression levels of STC1, ITGA2, TIMP3, and INHBA mainly resulted in the dysfunction of dNK cells in maternal–fetal tolerance during *T. gondii* infection. This result might help explain the molecular mechanism underlying dNK dysfunction following *T. gondii* infection. Additionally, the functions of DEGs were analyzed through GO enrichment. Interestingly, our results showed that the downregulated genes in dNK cells might be involved in regulating the TGF-β signaling pathway, whereas the upregulated genes might participate in the TNF signaling pathway following *T. gondii* infection. Although the production of TGF-β benefits the function of maternal–fetal immune tolerance [30, 31], the upregulation of TNF-α is responsible for the cytotoxicity function of dNK [6]. Our present results further indicated that during *T. gondii* infection, the function of maternal–fetal immune tolerance was weakened due to inhibiting TGF-β signaling pathway, whereas cytotoxicity function was enhanced resulting from the activating of TNF signaling pathway. Next, we analyzed the DEGs in the dNK subsets (NCAM1^+^FCGR3A^−^dNK and NCAM1^+^FCGR3A^+^dNK) after infection. Our results showed that there were 437 genes upregulated and 23 genes downregulated in NCAM1^+^FCGR3A^+^dNK, while 267 genes upregulated and 376 genes downregulated in NCAM1^+^FCGR3A^−^dNK inducing by *T. gondii* infection. Such changes may disrupt the balance between the NCAM1^+^FCGR3A^+^dNK and NCAM1^+^FCGR3A^−^dNK subsets, eventually resulting in the dysfunction of dNK cells after *T. gondii* infection. Interestingly, we found that the proinflammatory gene IFNG was significantly highly expressed in NCAM1^+^FCGR3A^+^dNK during *T. gondii* infection. Evidence has shown that CD56^+^CD16^+^dNK (NCAM1^+^FCGR3A^+^dNK) exhibited more robust response on the expression of IFNG [32]. Our results indicated that the cytotoxicity function of NCAM1^+^FCGR3A^+^dNK was enhanced due to IFNG overexpression during *T. gondii* infection, which contributed to adverse pregnancy outcomes. Through GO enrichment analysis, we also found that the functions of some enriched upregulated genes in NCAM1^+^FCGR3A^+^dNK cells were related to the NF-κβ signaling pathway. This result provided evidence showing that the occurrence of abnormal pregnancy outcome with *T. gondii* infection might be related to the activation of the NF-κβ signaling pathway in NCAM1^+^FCGR3A^+^dNK cells.

Furthermore, the changes of transcription factor in dNK cells after *T. gondii* infection were analyzed with SCENIC. Our results showed that the TFs, IRF8, and JUND were significantly upregulated. The above results were further validated with RT-PCR. Studies have reported that IRF8 could promote the expression of the inflammatory cytokines IFN-γ and IFN-β and that JUND was associated with inflammatory diseases [33–35]. Our results demonstrated that some inflammatory cytokines, such as IFN- γ and IFN-β, may be overproduced due to the upregulation of some inflammatory TFs, eventually enhancing the killing function of dNK during *T. gondii* infection.

Moreover, we analyzed the function of the target genes regulated by active TFs through KEGG and GO enrichment analyses. Our results suggested that during *T. gondii* infection, these target genes might be involved in the activation of the TNF and IFN-γ signaling pathways and cellular ubiquitination. Our previous study revealed that after *T. gondii* infection, IFN-γ and TNF-α are overproduced, leading to the dysfunction of dNK cells [6]. In this study, our results demonstrated that during *T. gondii* infection, several target genes were regulated by upregulated TFs, leading to the overproduction of IFN-γ and TNF-α. The result of KEGG and GO analyses in the present study showed that the ubiquitination of some target genes induced by infection might be regulated by upregulated TFs. Studies have proven that abnormal ubiquitin-specific protease might lead to aberrant trophoblast invasion, thereby contributing to recurrent miscarriages [36, 37]. Our results indicated that upregulated TFs cause the abnormal ubiquitination procession of some target genes in dNK cells and that this effect might be the important molecular mechanism of adverse pregnancy outcomes induced by *T. gondii* infection.

Studies have shown that dMφ cells account for approximately 10–20% of decidual leukocytes during the first trimester of pregnancy and participate in implantation, trophoblast invasion, and fetal development during pregnancy [38]. Early studies performed by our group have substantiated that the dysfunction of dMφ induced by *T. gondii* infection contributes to adverse pregnancy outcomes [7, 39, 40]. In this study, the heterogeneity of dMφ cells during *T. gondii* infection were analyzed through scRNA-seq. Our data revealed that the number of dMφ cells were decreased after *T. gondii* infection. This result was further validated with flow cytometry. DEG analysis revealed that 312 genes were differentially expressed in dMφ after *T. gondii* infection. These DEGs included 256 upregulated and 56 downregulated genes. Our results showed that numerous potential genes changed during *T. gondii* infection and provided numerous important key molecules that require further exploration and help explain the mechanism of adverse pregnancy outcomes. In particular, TNFSF13 and VSIG4 were significantly downregulated after *T. gondii* infection and were further validated via RT-PCR. Studies have reported that VSIG4 and TNFSF13 participate in immunosuppression via diverse immunoregulation pathways [41, 42]. Therefore, our results indicated that the maternal–fetal tolerance function of dMφ might be weakened due to the downregulation of VSIG4 and TNFSF13 during *T. gondii* infection. Furthermore, we analyzed the function of DEGs through GO enrichment. Interestingly, our results revealed that the genes in dMφ cells that were upregulated during *T. gondii* infection might participate in the signaling pathways related to the NOD-like receptor.

Additionally, SCENIC analysis revealed that after *T. gondii* infection, the TF IRF7 was mostly upregulated in dMφ and that the target genes it regulates were related to neutrophil degranulation and IFN-γ-mediated and type I interferon signaling pathways. IRF7 is involved in the inflammatory response in macrophages and is associated with M1 polarization [43]. Our results provided evidence showing that *T. gondii* infection may result in M1 polarization, which enhances the inflammatory response in dMφ due to the upregulation of the IRF7.

Next, we further analyzed the DEGs in dMφ subsets (TNF^+^dMφ, CD86^+^dMφ, CD163^+^dMφ, and TGFβ^+^dMφ) after infection. Our results showed that after *T. gondii* infection, the proportions of CD86^+^dMφ and CD163^+^dMφ in the total dMφ were all decreased, and the ratio of CD86^+^dMφ/CD163^+^dMφ was decreased. These changes demonstrated that *T. gondii* infection could promote M1 polarization. We also found that after *T. gondii* infection, the proportion of TGFβ^+^dMφ decreased, whereas that of TNF^+^dMφ increased. Our results indicated that due to *T. gondii* infection, the maternal–fetal tolerance function of dMφ weakened, whereas killing activity enhanced. Furthermore, we analyzed DEGs through scRNA-seq. Our results demonstrated that GBP1, GBP5, and IFIT3 were upregulated in the above four dMφ subsets after *T. gondii* infection. Previous studies have reported that the upregulation of GBP1, GBP5, and IFIT3 in Mφ could enhance the inflammatory reaction, induce apoptosis, or promote M1 polarization [44, 45]. We deduced from our scRNA-seq results that the cytotoxicity function of dMφ increased and M1 polarization strengthened during *T. gondii* infection.

Our scRNA-seq results showed that VSIG4 was downregulated in dMφ after *T. gondii* infection. The validation results of RT-PCR, immunofluorescence analysis, and flow cytometry were consistent with scRNA-seq data. VSIG4 acted as a co-inhibitory ligand that is specifically expressed in tissue-resident macrophages [21, 22]. Promoting VSIG4 expression greatly inhibited the release of proinflammatory factors (iNOS, CD16, and CD11b) while promoting the production of antiinflammatory mediators (Arg-1, IL-10, and CD206) [41]. These results indicated that the killing activity of dMφ due to iNOS overexpression was boosted, but the immune tolerance function declined due to the low Arg-1 and IL-10 production regulated by the *T. gondii*-induced reduction in VSIG4*.* Interestingly, KEGG pathway enrichment analysis showed that during *T. gondii* infection, the function of the downregulated genes in VSIG4^+^dMφ was associated with the TGF-β signaling pathway. Some studies have reported that TGF-β plays an essential role in maintaining fetal–maternal tolerance [30, 31]. Our previous study revealed that TGF-β1 treatment could improve the abnormal pregnancy outcomes with *T. gondii* infection by decreasing the cytotoxicity of dNK cells [46]. Therefore, our results suggested that the downregulation of VSIG4 might regulate the expression of TGF-β, resulting in the dysfunction of dMφ during *T. gondii* infection. Next, we used a VSIG4-neutralized antibody to further explore whether VSIG4 downregulation could decrease the expression of TGF-β in dMφ after infection. This speculation was validated through western blot analysis.

Studies have shown that decidual T cells comprise approximately 10–20% of decidual leukocytes during the first trimester of pregnancy and play important roles in normal and pathological pregnancies [3]. In the present study, the heterogeneity of dT cells during *T. gondii* infection through scRNA-seq. The analysis of DEGs in dT cells showed that among the 380 DEGs induced by *T. gondii* infection, 205 were upregulated and 175 were downregulated. Our work revealed that numerous unknown genes changed in dT during *T. gondii* infection and provided potential key molecules for further exploration to elucidate the mechanism of adverse pregnancy outcomes. The significantly downregulated genes (TIMP3, STC1, ITGA2, and TGFΒ1) induced by *T. gondii* infection identified through scRNA-seq were further validated through RT-PCR. Additionally, the function of DEGs was analyzed through GO enrichment analysis. Notably, our results showed that the downregulated genes in dT cells might be involved in regulating TGF-β, whereas the upregulated genes may participate in the TNF signaling pathway following *T. gondii* infection. Studies have shown that TGF-β production is beneficial to the function of maternal–fetal immune tolerance, whereas TNF-α upregulation is responsible for the cytotoxicity function of dT [47]. Our present results further indicated that during *T. gondii* infection, the weakened function of maternal–fetal immune tolerance might be associated with the inhibition of the TGF-β signaling pathway, whereas the enhancement in the cytotoxicity function was related to the activation of the TNF signaling pathway. Moreover, the heterogeneity of the TFs in dT after *T. gondii* infection was analyzed using SCENIC analysis. Interestingly, KEGG analysis revealed that the target genes regulated by M3 and M2 TFs might participate in TNF-mediated signaling pathways during *T. gondii* infection. The regulon of M2 also participated in the PD1–PDL1-related signaling pathways induced by *T. gondii* infection. Our previous studies have found that *T. gondii* infection could change the expression of PD-1 in dTreg cells and impair dTreg cell function, thus contributing to adverse pregnancy outcomes [48]. These results revealed that *T. gondii* infection could change the expression of several target genes in dT, leading to dysfunction by regulating the TNF signaling pathway and disrupting PD1–PDL1 interactions.

Maintaining the homeostasis of the microenvironment of the decidual immune cells at the maternal–fetal interface is essential for successful pregnancy [49]. Evidence has shown that communication among several decidual immune cells plays an important role in the maintenance of immune homeostasis [50]. We analyzed the intercellular receptor–ligand pairs and molecular interactions of immune cells using the CellPhoneDB algorithm (NOR versus INF) to observe the communication among the five types of human decidual immune cells (dNK, dMφ, dB, dT, and dDC). Our results revealed that after *T. gondii* infection, the strength and number of the receptor–ligand interactions among the decidual immune cells decreased. Therefore, these results indicated that communication among decidual immune cells maintaining immune homeostasis weakened after *T. gondii* infection. This effect might be harmful to maternal–fetal immune tolerance. We further analyzed the diverse receptor–ligand interplays of decidual immune cells during *T. gondii* infection. Notably, the strength of some immune inhibitory receptor–ligand interactions, such as TNFSF13–TNFSF13B, TIGIT–PVR, TIGIT–NECTIN2, HLA-E:CD94–NKG2, and LGALS9–HAVCR2, weakened, whereas some immune active interactions, such as MIF–(CD74 + CXCR4) and CD80–CD274, enhanced after *T. gondii* infection. This result revealed that the maternal–fetal immune tolerance function declined, whereas the killing ability of decidual immune cells enhanced due to communication changes, eventually contributing to the occurrence of adverse pregnancy outcomes.

## Conclusions

The proportion of 17 subsets in decidual immune cells and the expression level of 21 genes were changed after *T. gondii* infection. The expression levels of multiple genes and transcription factors were downregulated or upregulated induced by *T. gondii* infection. Upregulated genes were related to NOD-like receptor signaling pathways in dMφ, TNF signaling pathways in dNK, dMφ and dT, IFN-γ and type I interferon signaling pathways in dNK after *T. gondii* infection. Downregulated genes were related to TGF-β signaling pathway in dNK and dT. The expression level of VSIG4 on dMφ were downregulated after *T. gondii* infection, which further regulate TGF-β expression. The interaction among dNK, dMφ, dDC, dB, dT was changed during *T. gondii* infection. Our present study might accelerate the exploration of the immune molecular mechanism of adverse pregnancy outcomes due to *T. gondii* infection.

### Supplementary Information

**Additional file 1: Fig. S1.** The re-clusters of decidual immune cells (PTPRC^+^). **a** The UMAP map of immune cells and non-immune cells from human decidual tissue. **b** The profile of decidual non-immune cells (COL1A1^+^ or COL1A2^+^ or DCN^+^) and immune cells (PTPRC^+^).** c** Heatmap of representative marker genes in each cluster of decidual immune cells.

**Additional file 2: Fig. S2.** The major subsets of decidual immune cells. The expression profile of decidual natural killer cells (dNK, NCAM1 positive), decidual macrophages (dMφ, CD14, or CD68 positive), decidual T cells (dT, CD3D, CD4, CD8A, and CD8B positive), decidual B cells (dB, CD79A positive) and decidual dendritic cells (dDC, HLA-DRA positive).

**Additional file 3: Fig. S3.** The representative makers of decidual macrophage subsets. **a** The representative makers of TGFB1^+^dMφ subset. **b** The representative makers of CD163^+^dMφ subset. **c** The representative makers of CD86^+^dMφ. **d** The representative makers of TNF^+^dMφ subset.

**Additional file 4: Fig. S4**. The expression profile of VSIG4 in dNK, dMφ, and dDC. **a** The expression percentages of VSIG4 on dNK by flow cytometry. **b** The expression percentages of VSIG4 on dMφ by flow cytometry. **c** The expression percentages of VSIG4 on dDC by flow cytometry.

**Additional file 5: Fig. S5.** Significant signaling pathways of cell–cell connections in decidual immune cell subsets. IL-2 **a**, CD80 **b **, and TIGIT **c **signaling pathway network of dNK, dMφ, dT, dB, and dDC subsets between NOR and INF groups.

**Additional file 6: Table S1.** Primers used in this study.

## Data Availability

All the data generated in this study are presented within the published article. The RNA-seq raw data described in the present study has been submitted to the GEO database. Accession number is GSE254302.

## Abbreviations

dT: Decidual T cells
dMφ: Decidual macrophages
dNK: Decidual natural killer cells
FBS: Fetal bovine serum
HLA-DR: Human leukocyte antigen DR
APC: Allophycocyanin
FITC: Fluorescein isothiocyanate
PE: Phycoerythrin
PBS: Phosphate buffered saline
RT-PCR: Reverse transcriptase polymerase chain reaction
DEGs: Differentially expressed genes
PBMC: Peripheral blood mononuclear cell
KEGG: Kyoto Encyclopedia of Genes and Genomes
GO: Gene ontology
TF: Transcription factor
