# Supplementary material for: Single-cell transcriptomic analysis of decidual immune cell landscape in the occurrence of adverse pregnancy outcomes induced by Toxoplasma gondii infection
Source: Parasit Vectors. 2024 May 10;17:213. doi: 10.1186/s13071-024-06266-w (PMC11088043; doi:10.1186/s13071-024-06266-w)
Supplement: Supplementary file 1 — Additional file 1: Fig. S1. The re-clusters of decidual immune cells (PTPRC+). a The UMAP map of immune cells and non-immune cells from human decidual tissue. b The profile of decidual non-immune cells (COL1A1+ or COL1A2+ or DCN+) and immune cells (PTPRC+). c Heatmap of representative marker genes in each cluster of decidual immune cells. [file 13071_2024_6266_MOESM1_ESM.docx]

**Single-cell transcriptomic analysis of decidual immune cell landscape in the occurrence of adverse pregnancy outcomes induced by** ***Toxoplasma gondii* infection**

Tianyi Fu^1#^, Xiaohui Wang^1#^, Xiaoyue Zhao^2#^, Yuzhu Jiang^1^, Xianbing Liu^1^, Haixia Zhang^1^, Yushan Ren^1^, Zhidan Li^1*^, Xuemei Hu^1*^

**Supplementary information**

**
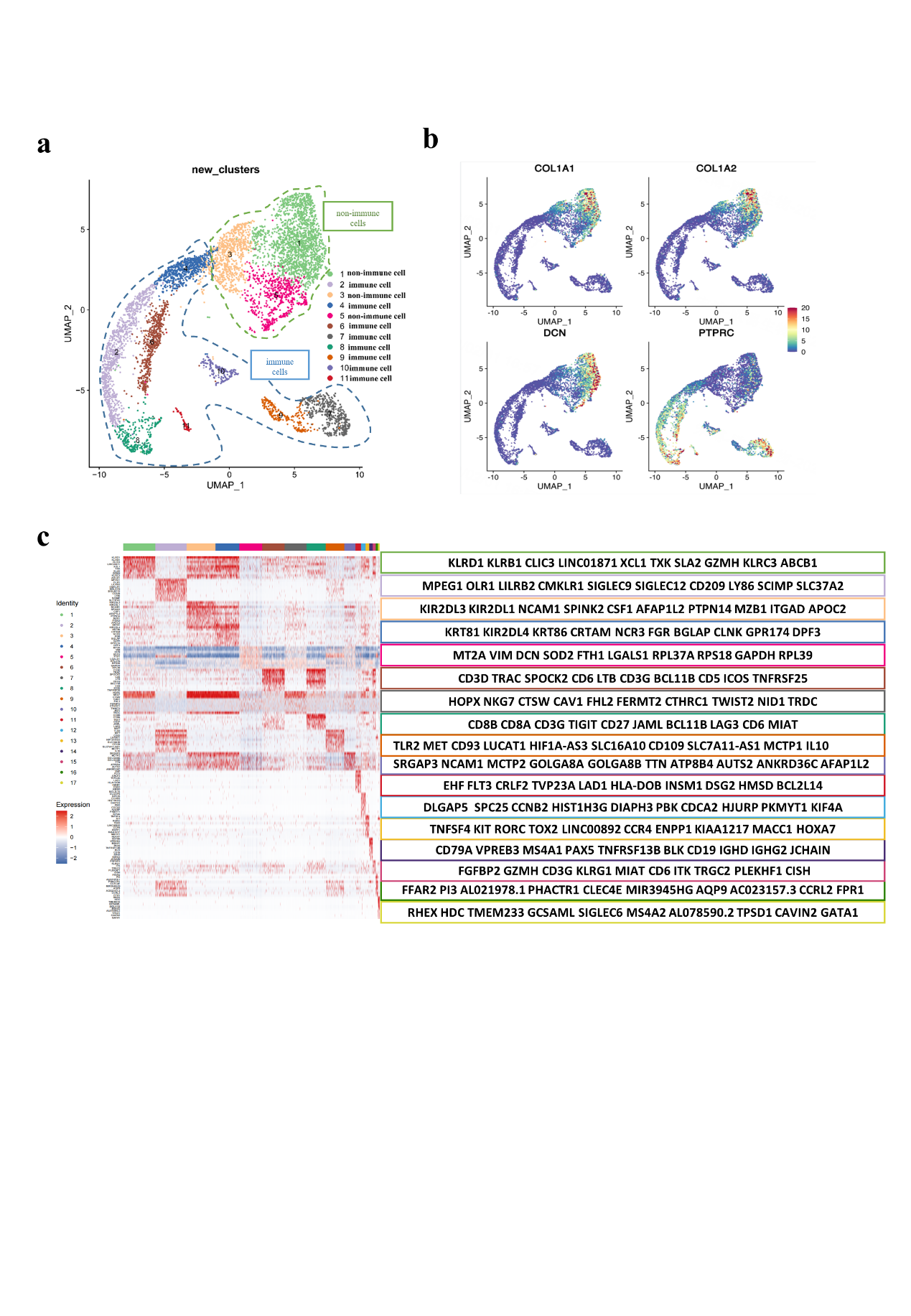
**

**Additional file 1: Fig. S1.** The re-clusters of decidual immune cells (PTPRC^+^). **a** The UMAP map of immune cells and non-immune cells from human decidual tissue. **b** The profile of decidual non-immune cells (COL1A1^+^ or COL1A2^+^ or DCN^+^) and immune cells (PTPRC^+^). **c** Heatmap of representative marker genes in each cluster of decidual immune cells.
