# Supplementary material for: Single-cell transcriptomic analysis of decidual immune cell landscape in the occurrence of adverse pregnancy outcomes induced by Toxoplasma gondii infection
Source: Parasit Vectors. 2024 May 10;17:213. doi: 10.1186/s13071-024-06266-w (PMC11088043; doi:10.1186/s13071-024-06266-w)
Supplement: Supplementary file 2 — Additional file 2: Fig. S2. The major subsets of decidual immune cells. The expression profile of decidual natural killer cells (dNK, NCAM1 positive), decidual macrophages (dMφ, CD14, or CD68 positive), decidual T cells (dT, CD3D, CD4, CD8A, and CD8B positive), decidual B cells (dB, CD79A positive) and decidual dendritic cells (dDC, HLA-DRA positive). [file 13071_2024_6266_MOESM2_ESM.docx]

**
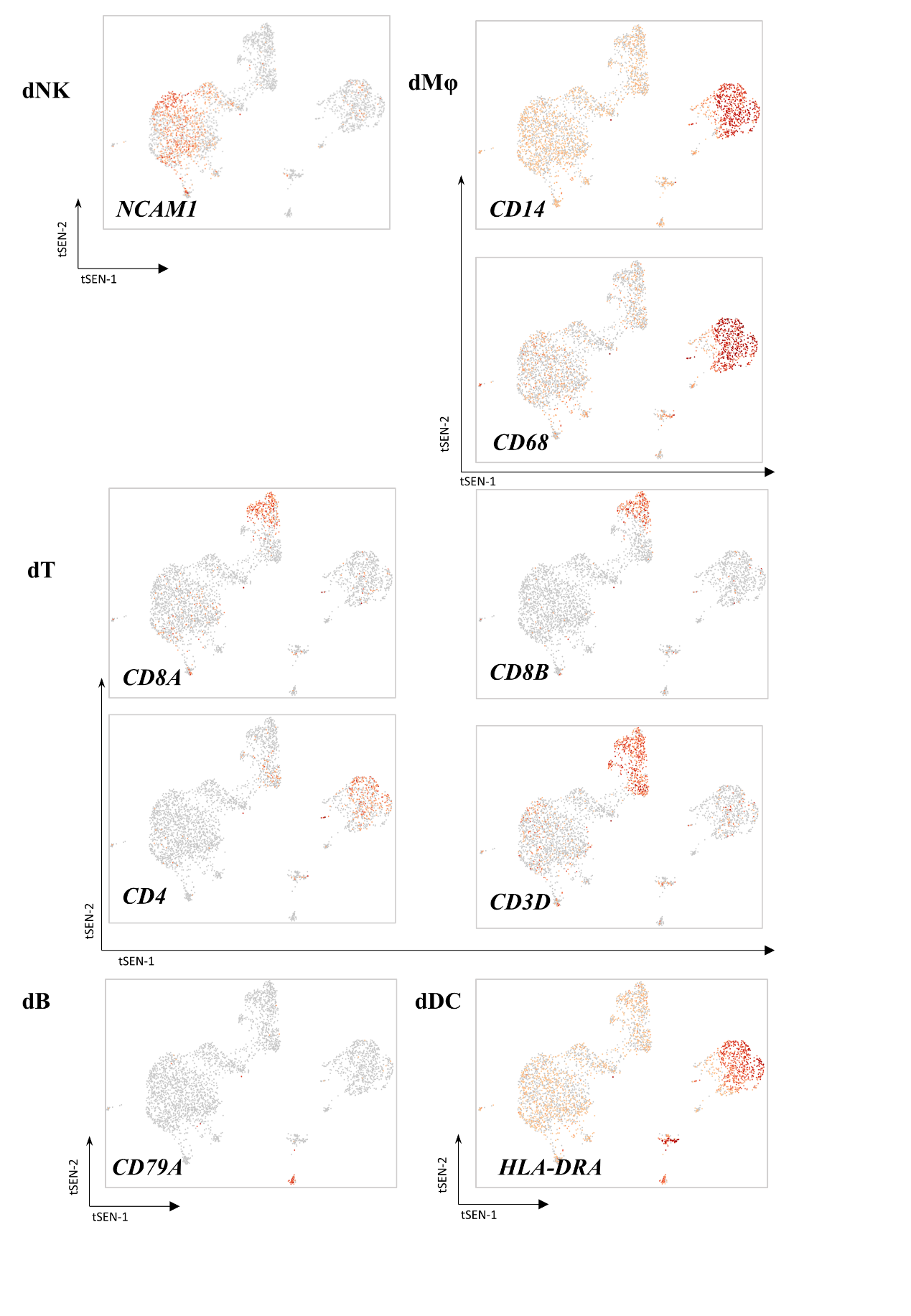
**

**Additional file 2: Fig. S2.** The major subsets of decidual immune cells. The expression profile of decidual natural killer cells (dNK, NCAM1 positive), decidual macrophages (dMφ, CD14 or CD68 positive), decidual T cells (dT, CD3D, CD4，CD8A and CD8B positive), decidual B cells (dB, CD79A positive) and decidual dendritic cells (dDC, HLA-DRA positive).
