# Supplementary material for: Single-cell transcriptomic analysis of decidual immune cell landscape in the occurrence of adverse pregnancy outcomes induced by Toxoplasma gondii infection
Source: Parasit Vectors. 2024 May 10;17:213. doi: 10.1186/s13071-024-06266-w (PMC11088043; doi:10.1186/s13071-024-06266-w)
Supplement: Supplementary file 3 — Additional file 3: Fig. S3. The representative makers of decidual macrophage subsets. a The representative makers of TGFB1+dMφ subset. b The representative makers of CD163+dMφ subset. c The representative makers of CD86+dMφ. d The representative makers of TNF+dMφ subset. [file 13071_2024_6266_MOESM3_ESM.docx]

**
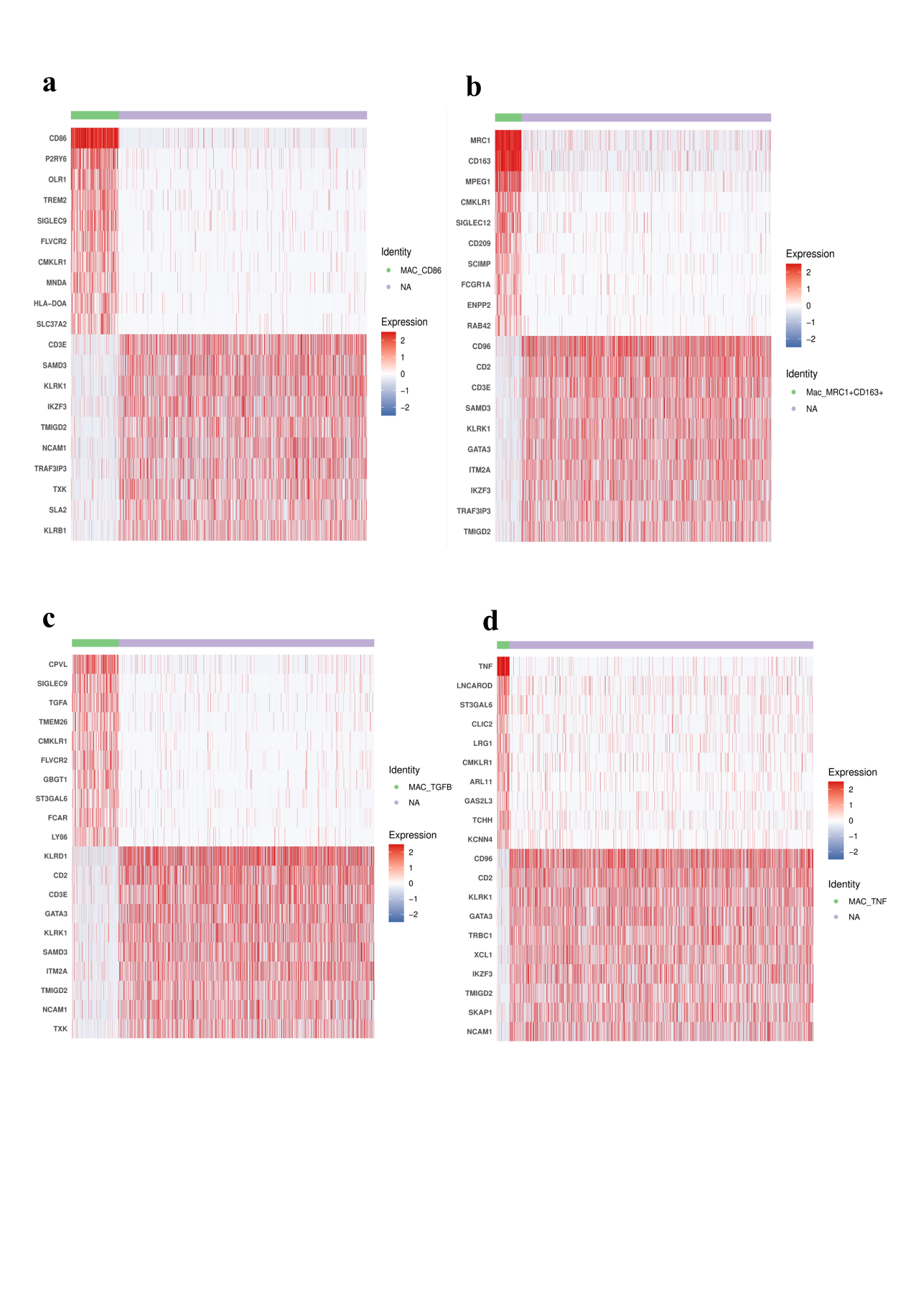
**

**Additional file 3: Fig. S3.** **The representative makers of decidual macrophage subsets. a** The representative makers of TGFB1^+^dMφ subset. **b** The representative makers of CD163^+^dMφ subset. **c** The representative makers of CD86^+^dMφ. **d** The representative makers of TNF^+^dMφ subset.
