# Supplementary material for: Single-cell transcriptomic analysis of decidual immune cell landscape in the occurrence of adverse pregnancy outcomes induced by Toxoplasma gondii infection
Source: Parasit Vectors. 2024 May 10;17:213. doi: 10.1186/s13071-024-06266-w (PMC11088043; doi:10.1186/s13071-024-06266-w)
Supplement: Supplementary file 4 — Additional file 4: Fig. S4. The expression profile of VSIG4 in dNK, dMφ, and dDC. a The expression percentages of VSIG4 on dNK by flow cytometry. b The expression percentages of VSIG4 on dMφ by flow cytometry. c The expression percentages of VSIG4 on dDC by flow cytometry. [file 13071_2024_6266_MOESM4_ESM.docx]

**
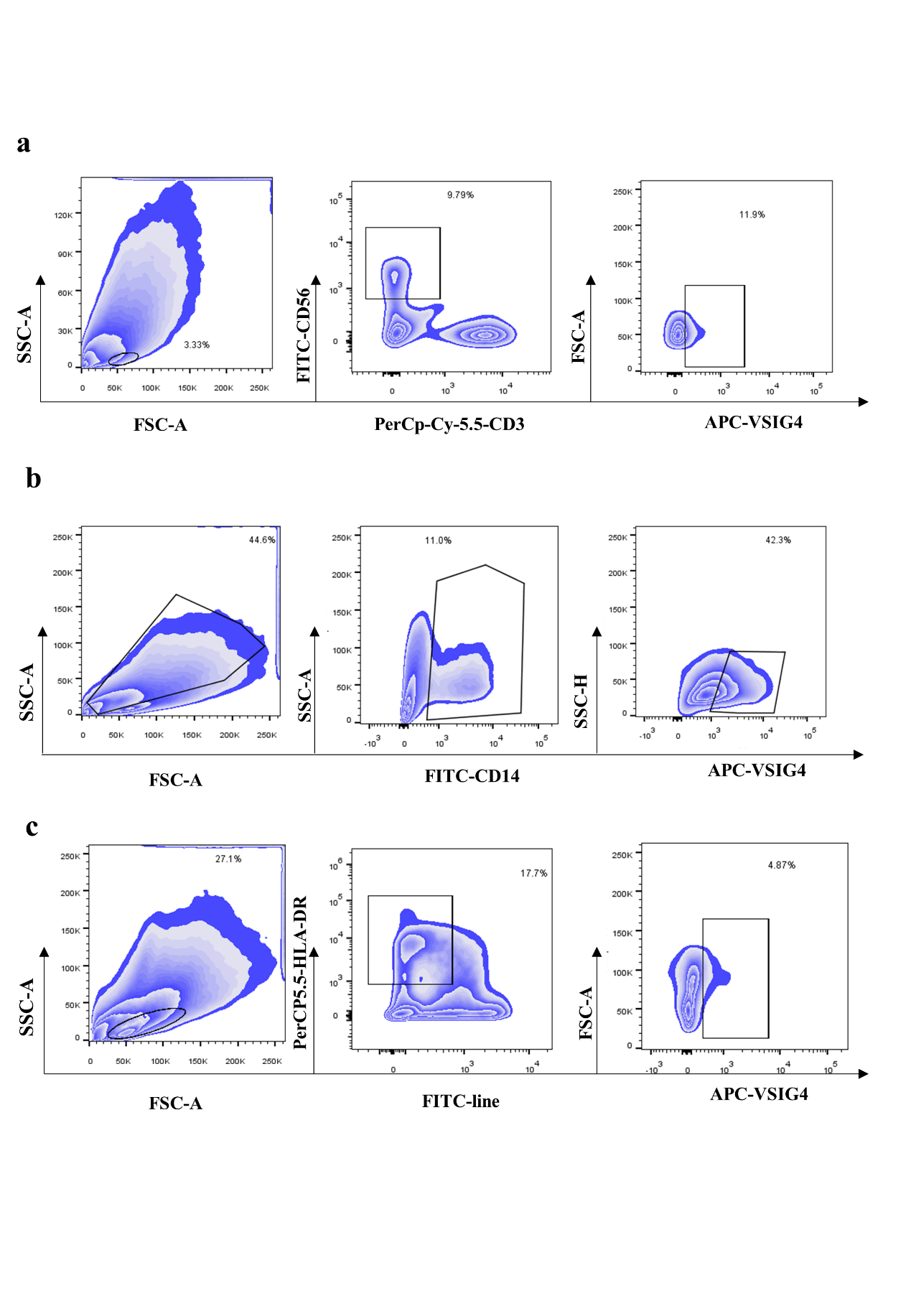
**

**Additional file 4: Fig. S4.** The expression profile of VSIG4 in dNK, dMφ and dDC. **a** The expression percentages of VSIG4 on dNK by flow cytometry **b** The expression percentages of VSIG4 on dMφ by flow cytometry. **c** The expression percentages of VSIG4 on dDC by flow cytometry.
