# Supplementary material for: Single-cell transcriptomic analysis of decidual immune cell landscape in the occurrence of adverse pregnancy outcomes induced by Toxoplasma gondii infection
Source: Parasit Vectors. 2024 May 10;17:213. doi: 10.1186/s13071-024-06266-w (PMC11088043; doi:10.1186/s13071-024-06266-w)
Supplement: Supplementary file 5 — Additional file 5: Fig. S5. Significant signaling pathways of cell–cell connections in decidual immune cell subsets. IL-2 a, CD80 b , and TIGIT c signaling pathway network of dNK, dMφ, dT, dB, and dDC subsets between NOR and INF groups. [file 13071_2024_6266_MOESM5_ESM.docx]

**
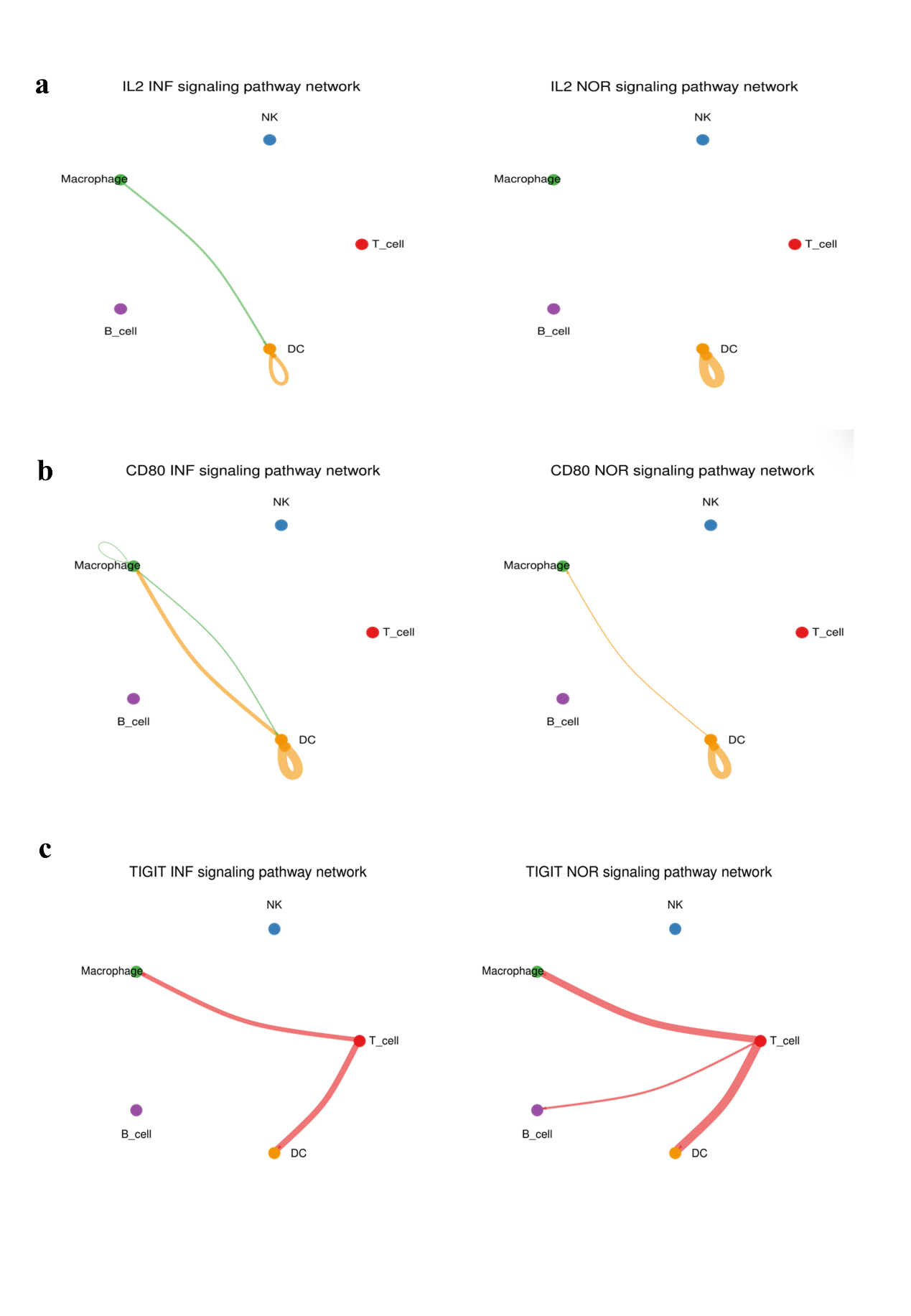
**

**Additional file 5: Fig. S5.** Significant signaling pathways of cell–cell connections in decidual immune cell subsets. IL-2 **a**, CD80 **b** and TIGIT **c** signaling pathway network of dNK, dMφ, dT, dB and dDC subsets between NOR and INF groups.
