# Supplementary material for: Single-cell transcriptomic analysis of decidual immune cell landscape in the occurrence of adverse pregnancy outcomes induced by Toxoplasma gondii infection
Source: Parasit Vectors. 2024 May 10;17:213. doi: 10.1186/s13071-024-06266-w (PMC11088043; doi:10.1186/s13071-024-06266-w)
Supplement: Supplementary file 6 — Additional file 6: Table S1. Primers used in this study. [file 13071_2024_6266_MOESM6_ESM.docx]

**Additional file 6: Table S1.** Primers used in this study.

| **Gene name** | **Forward primer** | **Reverse primer** |
| --- | --- | --- |
| STC1 | F:5′-ATTCCCACCAACAAAATCCA-3′ | R:5′-GGAAAAACATGGCAGAGGAA-3′ |
| ITGA2 | F:5′-CTCTCAGCCAGCTTCTCACC-3′ | R:5’-ACCCCACCTGTGTCTTTGTG-3′ |
| TIMP3 | F:5′-AGTACATCCACACGGAAG-3′ | R:5′-AAGCAAGGCAGGTAGTAG-3′ |
| INHBA | F:5′-GTCGCACAGACCTTTCCTCA-3′ | R:5′-CCAGTCATTCCAGCCGATGT-3′ |
| IRF8 | F:5′-TCTTCGACACCAGCCAGTTC-3′ | R:5’-CAGCTCTTCCCAGCCTCTTC-3′ |
| JUND | F:5′-GCGCCTGGAAGAGAAAGTGA-3′ | R:5′-TGACGTGGCTGAGGACTTTC-3′ |
| VSIG4 | F:5′-ATGGGGATCTTACTGGGCCT-3′ | R:5′-GTCTGAGCCACGTTGTACCA-3′ |
| TNFSF13 | F:5′-TTGCCCTCTGGTTGAGTTGG-3′ | R:5’-CTGGTTGCCACATCACCTCT-3′ |
| IRF7 | F:5′-TCAGACATTGGGAGCAGCAG-3′ | R:5′-TGAAGGTGAGCAGCAGTGAG-3′ |
| TGFΒ1 | F:5′-ATGACAAGTTCAAGCAGAG-3′ | R:5’-CACTTGCAGTGTGTTATCC-3′ |
| GAPDH | F: 5′-GGAGCGAGATCCCTCCAAAAT-3′ | R:5′-GCTGTTGTCATACTTCTCATGG-3′ |
